# mRNA-based vaccines and therapeutics: an in-depth survey of current and upcoming clinical applications

**DOI:** 10.1186/s12929-023-00977-5

**Published:** 2023-10-07

**Authors:** Yu-Shiuan Wang, Monika Kumari, Guan-Hong Chen, Ming-Hsiang Hong, Joyce Pei-Yi Yuan, Jui-Ling Tsai, Han-Chung Wu

**Affiliations:** 1 Institute of Cellular and Organismic Biology, Academia Sinica, No. 128, Academia Road, Section 2, Nankang, Taipei 11529 Taiwan; 2 Biomedical Translation Research Center (BioTReC), Academia Sinica, Taipei, 11571 Taiwan

**Keywords:** mRNA vaccine, mRNA therapeutics, Lipid nanoparticles, Targeting mRNA delivery system, Administration routes

## Abstract

mRNA-based drugs have tremendous potential as clinical treatments, however, a major challenge in realizing this drug class will promise to develop methods for safely delivering the bioactive agents with high efficiency and without activating the immune system. With regard to mRNA vaccines, researchers have modified the mRNA structure to enhance its stability and promote systemic tolerance of antigenic presentation in non-inflammatory contexts. Still, delivery of naked modified mRNAs is inefficient and results in low levels of antigen protein production. As such, lipid nanoparticles have been utilized to improve delivery and protect the mRNA cargo from extracellular degradation. This advance was a major milestone in the development of mRNA vaccines and dispelled skepticism about the potential of this technology to yield clinically approved medicines. Following the resounding success of mRNA vaccines for COVID-19, many other mRNA-based drugs have been proposed for the treatment of a variety of diseases. This review begins with a discussion of mRNA modifications and delivery vehicles, as well as the factors that influence administration routes. Then, we summarize the potential applications of mRNA-based drugs and discuss further key points pertaining to preclinical and clinical development of mRNA drugs targeting a wide range of diseases. Finally, we discuss the latest market trends and future applications of mRNA-based drugs.

## Introduction

An mRNA molecule consists of a single-stranded ribonucleic acid that carries coding information for the translation and processing of functional proteins [141, 164]. Early studies on mRNA showed that the molecules can also play multifunctional roles in regulating gene function [245]. Due to its potential therapeutic utility, numerous mRNA-based therapies have been proposed. The first study reported in vivo study on mRNA-based drugs involved injecting mice in skeletal muscle with naked mRNAs to stimulate the expression of the functional proteins [252]. Subsequently, mRNA-based drugs have emerged as an attractive new therapeutic class, which is expected to revolutionize cancer treatment through different approaches, such as therapeutic vaccines, monoclonal antibodies, immunomodulatory drugs, and chimeric antigen receptor (CAR) cell therapy [22, 62, 73, 164, 228]. Compared to other functional biomolecules such as DNA plasmids and recombinant proteins, mRNA has multiple therapeutic advantages that make it ideal for the development of next-generation cancer immunotherapy drugs. One major difference between mRNA- and DNA-based drugs is that mRNAs can theoretically serve as templates for the production of any protein/peptide by utilizing the protein synthesis process in transfected cells [101]. In addition, mRNA-based drugs have higher transfection efficiencies and lower toxicities than DNA-based drugs, and they do not require translocation into the nucleus to function [164]. Moreover, mRNA molecules are not prone to insertional mutagenesis and thus carry a reduced risk of accidental infection [153]. Due to the continuous translation of mRNA templates, which leads to persistent expression of encoded proteins/peptides, mRNAs have a greater potential for the treatment of diseases requiring high protein levels with higher therapeutic effectiveness [48]. A previous study by our lab demonstrated that mRNA-lipid nanoparticle (mRNA-LNP) technology could be used to successfully produce monoclonal antibodies (mAbs) against the receptor binding domains (RBDs) of SARS-CoV-2 spike (S) proteins from different variants [92].

Recent studies have reported many desirable features of mRNA-based approaches that aid in the drug development process [34]. First, mRNAs can be easily designed and modified by generating appropriate genomic sequences,then, the drugs can be rapidly synthesized using in vitro transcription (IVT) technology. Second, mRNAs can mediate the transient expression of therapeutically functional proteins without any risk of genomic integration [22]. In addition, it is possible to expand the scope of successful treatment to other disease states, as subsequent mRNA drugs can be rapidly derived from existing technologies by simply changing the template DNA sequence. However, there are serval critical issues that must be addressed during the development of mRNA-based drugs: (1) delivery of the therapeutic mRNA to target cells should be maximally effective, (2) successful mRNA translation to functional protein must be confirmed, and (3) potential immunogenicity of the mRNA should be minimized. Regarding the issue of delivery, numerous non-viral delivery systems have been tested and applied to overcome the natural susceptibility of mRNA to enzymatic degradation [220, 225, 242]. The successful development of mRNA-based COVID-19 vaccines has opened up exciting new opportunities for novel mRNA-based drugs and vaccines to combat a variety of challenging diseases. Some of the most promising applications of mRNA-based drugs and vaccines currently in clinical trials are outlined in Table 1. As mRNA-based drug design continues to improve, it is important to understand the current state and trajectory of mRNA engineering and translation efficacy. In this review, we not only discuss the different types of mRNA used for mRNA-based vaccines and drugs, but also describe the current progress in optimizing mRNA delivery, including a discussion of pre-clinical, clinical, and FDA-approved modalities. In addition, we delineate the advantages and potential applications of using mRNA to treat different diseases and provide perspectives on mRNA-based therapeutics in future clinical applications.

**Table 1:** Overview of ongoing mRNA-based drug’s clinical trials

Trial number	Delivery system/route	mRNA encoding	Product name	Disease/virus	Phase	Start date (MM/YYYY)
COVID-19						
NCT04776317	SAM-LNP, i.m	SARS-CoV-2 spike	NR	COVID-19	I	03/2021
NCT04805125	LNP, i.m	SARS-CoV-2 spike	mRNA-1273	COVID-19	III	04/2021
NCT04824638	LNP, i.m	SARS-CoV-2 spike	BNT162b2	COVID-19	II	03/2021
NCT04900467	LNP, i.m	SARS-CoV-2 spike	BNT162b2/mRNA-1273	COVID-19	NR	05/2021
NCT04961229	LNP, i.m	SARS-CoV-2 spike	BNT162b2	COVID-19	IV	10/2021
NCT05124171	LNP, i.m	SARS-CoV-2 spike	BNT162b2	COVID-19	III	12/2021
NCT05428592	LNP, i.m	SARS-CoV-2 spike	LVRNA009	COVID-19	III	04/2023
NCT05549206	LNP, i.m	SARS-CoV-2 spike (Omicron BA.5)	LVRNA012	COVID-19	NR	04/2023
NCT05599802	LNP, i.m	SARS-CoV-2 spike variant	LVRNA010	COVID-19	I	02/2023
NCT05602961	LNP, i.m	SARS-CoV-2 spike	GLB-COV2-043	COVID-19	I/II	02/2023
NCT05609045	LNP, i.m	SARS-CoV-2 Omicron variant	RH109	COVID-19	I	06/2023
NCT05658523	LNP, i.m	SARS-CoV-2 spike	Moderna/Novavax	COVID-19	III	02/2023
NCT05672355	MVA, i.m	SARS-CoV-2 spike	GEO-CM04S1	COVID-19	II	09/2023
NCT05682638	LNP, i.m	SARS-CoV-2 spike	LVRNA009	COVID-19	III	01/2023
NCT05743335	LNP, i.m	SARS-CoV-2 spike	JCXH-221	COVID-19	I/II	03/2023
NCT05745545	LNP, i.m	SARS-CoV-2 spike (Omicron BA.5)	NR	COVID-19	NR	02/2023
NCT05749926	LNP, i.m	SARS-CoV-2 spike (Omicron BA.5)	BNT162b2/Sanofi	COVID-19	III	05/2023
NCT05788185	LNP, i.m	SARS-CoV-2 spike	RVM-V001/RVM-V002	COVID-19	I/II	03/2023
NCT05812014	LNP, i.m	SARS-CoV-2 spike	LVRNA021	COVID-19	III	03/2023
NCT05815498	LNP, i.m	SARS-CoV-2 spike	mRNA-1283.222	COVID-19	III	03/2023
NCT05827926	LNP, i.m	SARS-CoV-2 spike + HA	mRNA-1083	COVID-19/Influenza	I/II	04/2023
NCT05875701	LNP, i.m	SARS-CoV-2 spike	Novavax	COVID-19	III	03/2023
NCT05907044	LNP, i.m	SARS-CoV-2 spike (Alpha, Beta + Omicron XBB.1.5/Alpha, Beta + Omicron BA.2, 4, 5)	RQ3027/RQ3025	COVID-19	NR	05/2023
NCT05911087	LNP, i.m	SARS-CoV-2 spike	SWIM816	COVID-19	II/III	06/2023
Cancer						
mRNA-based vaccine						
NCT03897881	LNP, i.m	Neoantigen	mRNA-4157	Stage III/IV melanoma	IIb	07/2019
NCT03908671	LNP, s.c	Neoantigen	PCV	EC/NSCLC	I	10/2019
NCT04161755	LNP, i.v	Neoantigen	PCV	PC	I	12/2019
NCT04382898	LNP, i.v	4 cancer antigens	BNT112	mCRPC/LPC	II	12/2019
NCT04683939	LNP, i.v	CLDN18.2	BNT141	GC/PC/OC/BTC	II	01/2022
NCT05761717	LNP, s.c	Personalised cancer antigen	NR	Postoperative HC	NR	04/2023
NCT05738447	LNP, i.m	HBsAg	HBV vaccine	HC	I	02/2023
mRNA-based cell therapy						
NCT01197625	i.v	hTERT/Survivin	DC vaccine	PrCa	II	09/2010
NCT04503278	RNA-LPX, i.v	CLDN6	BNT211	Advanced solid tumors	I/IIa	09/2020
NCT04981691	i.v	MESO	Amaretto	Refractory malignant solid neoplasm	I	10/2021
NCT04984356	i.v	CD7	WU-CART-007	T-ALL/LBL	I/II	01/2022
RiboMab (bispecific monoclonal antibody (mAb)-encoding mRNA)						
NCT04683939	LNP, i.m	CLDN18.2	BNT141	Multiple solid tumors	I/IIa	01/2022
Infectious disease						
NCT04917861	LNP, i.m.	Zika	mRNA-1893	Flavivirus	II	06/2021
NCT05085366	LNP, i.m.	gB	mRNA-1647	CMV	III	10/2021
NCT05127434	LNP, i.m.	preF glycoprotein	mRNA-1345	RSV-LRTD	II/III	11/2021
NCT05164094	LNP, i.m.	EBV gB (gB/gH/gL/gp42/gB350)	mRNA-1189	EBV	I	12/2021
NCT05217641	LNP, i.m.	BG505 MD39.3/BG505 MD39.3 gp151/BG505 MD39.3 gp151 CD4KO	mRNA -1574	HIV	I	02/2022
NCT05398796	LNP, i.m.	pre-F/G	mRNA -1215	Nipah virus	I	07/2022
NCT05414786	LNP, i.p.	eOD-GT8 60mer	mRNA-1644	AIDS	I	05/2022
NCT05415462	LNP, i.m.	HA	mRNA-1010	Seasonal influenza	III	06/2022
NCT05683457	LNP, i.m.	gB	mRNA-1647	CMV	II	04/2023
NCT05701800	LNP, i.m.	zoster virus envelope glycoprotein E	mRNA-1468	Herpes zoster	I/II	01/2023
NCT05743881	LNP, i.m.	preF glycoprotein	mRNA-1345/mRNA-1365	RSV-LRTD	I	02/2023
NCT05755620	LNP, i.m.	HA	H1ssF-3928	Influenza	I	04/2023
NCT05823974	LNP, i.m.	NR	GSK4382276A	Influenza	I/II	04/2023
NCT05827068	LNP, i.m.	HA	mRNA-1011.1/mRNA-1011.2/mRNA-1012.1	Seasonal influenza	I/II	03/2023
NCT05827978	LNP, i.m.	HA	mRNA-1010	Seasonal influenza	III	04/2023
NCT05829356	LNP, i.m.	Full-length HA sequence of A/Tasmania/503/2020 (H3N2)	NR	Influenza	I	04/2023
NCT05831111	LNP, i.m.	EBV gB	mRNA-1195	EBV	I	04/2023
NCT05868382	LNP, i.m.	HA	mRNA-1010 candidate variations	Influenza	II	05/2023
NCT05905731	i.v.	HBV-TCR	NR	CHB	I	06/2023
Protein replacement						
NCT04442347	LNP, i.v.	OTC	ARCT-810	OTCD	Ib	
Gene editing						
In vivo						
NCT04601051	LNP, i.v.	TTR-targeted CRISPR-Cas9	NTLA-2001	ATTRv-PN/ATTR-CM	I	11/2020
Ex vivo						
NCT03655678	i.v.	BCL11A-targeted gRNA	CTX001	TDT	III	09/2018
NCT04426669	i.v.	CISH-targeted CRISPR-Cas9	NR	GI cancer	I/II	05/2020
NCT05456880	i.v.	HBG1/2 promoter-targeted CRISPR-Cas9	BEAM-101	SCD	I/II	08/2022

*AIDS* acquired immunodeficiency syndrome, *ATTR-CM* transthyretin amyloidosis-related cardiomyopathy, *ATTRv-PN* hereditary transthyretin amyloidosis with polyneuropathy, *BTC* biliary tract cancer, *CHB* chronic hepatitis B, *CISH* cytokine-induced SH2 protein, *CLDN18.2* claudin-18.2, *CML* chronic myeloid leukemia, *CMV* cytomegalovirus, *EC* esophageal cancer, *EBV* epstein-barr virus, *gB* Glycoprotein B, *GC* gastric cancer, *GI* gastro-intestinal, *HA* hemagglutinin, *HBsAg* hepatitis B surface antigen, *HBV-TCR* hepatitis-B virus-antigen-specific T cell receptor, *HC* hepatocellular carcinoma, *HIV* human immunodeficiency virus, *i.m.* intramuscular, *i.p.* intraperitoneal, *i.v.* intravenous, *LBL* Lymphoblastic Lymphoma, *LPC* localized prostate cancer, *mCRPC* metastatic castration resistant prostate cancer, *MESO* mesothelin, *MVA* modified vaccinia virus Ankara, *NR* Not reported, *NSCLC* nonsmall cell lung cancer, *OC* ovarian cancer, *OTC* ornithine transcarbamylase, *OTCD* ornithine transcarbamylase deficiency disease, *PC* pancreatic cancer, *PrCa* prostate cancer, *pre-F/G* secreted prefusion stabilized F component covalently linked to G monomer, *RSV-LRTD* respiratory syncytial virus-associated lower respiratory tract disease, *SAM-LNP* self-amplifying mRNA-lipid nanoparticles, *S.C.* subcutaneous injection, *SCD* sickle cell disease, *T-ALL* T-cell acute lymphoblastic leukemia, *TDT* transfusion-dependent β-thalassemia, *TTR* transthyretin

## Synthesis and modification of mRNA vaccines and drugs

The structure of an mRNA molecule can be divided into several parts including a 5′ cap, 3′ poly (A) tail, 5′ and 3′ untranslated regions (5′- and 3′-UTRs), and an open reading frame (ORF). A number of these components can be altered to enhance the translatability or stability of mRNAs to make them suitable for clinical mRNA drugs [40, 90]. Below, we describe how mRNA compositions can be optimized for drug design.

### Cap structure

Eukaryotic RNA undergoes several modifications before being exported from the nucleus to cytosol for protein translation, the first of which is 5′ capping. Capping is needed to enhance mRNA stability, processing, export, and translation [64]. It is a three-step process mediated by RNA triphosphatase, guanylyltransferase, and methyltransferase, which yields a 7-methylguanosine (m7G) at the 5′ end of the mRNA followed by a triphosphate connection to the first nucleotide (m7GpppN, Cap0). This process occurs during transcription, and the three capping enzymes are coupled with RNA polymerase II [200]. The resultant 5′ cap then serves to regulate pre-mRNA splicing and nuclear export, protect RNA from exonuclease cleavage, and recruit translation initiation factors for protein production [43]. The 5′ cap structure also allows for differentiation between self and non-self mRNA molecules [49]. Thus, synthetic mRNAs with modified cap analogs should be generated to mimic fully processed mRNAs and avoid innate immune stimulation [42]. In total, there are four identified cap structures, namely cap 0, cap 1, cap 2, and the N6, 2′-*O*-dimethyladenosine (m6Am) cap. Cap 1 is formed by methylation of the 2ʹ-hydroxyl group on cap 0, and subsequent 2ʹ-*O*-methylation on the second nucleotide will form cap 2, which is present on ~ 50% of all transcripts. The cytosolic innate immune receptor Retinoic Acid Inducible Gene-I (RIG-I) recognizes uncapped RNAs or those with cap 0, but RNAs with cap 1 modifications escape recognition by RIG-I [49]. Although the majority of mRNAs possess cap 2 structures, the functional effects and molecular actions of mRNA cap 2 remain largely unclear. Meanwhile, the m6Am cap is formed by N6 methylation on the first adenosine nucleotide. Approximately, 30–40% of mRNAs possess an m6Am cap and may therefore be recognized by specific translation initiation factors to enhance translation [240]. In addition, m6Am-capped RNAs are known to exhibit relatively high stability in cells [142].

There are two methods currently used to manufacture capped RNAs. The first is to produce cap 0 or cap 1 RNAs using vaccinia virus capping enzymes; this method is comparatively expensive [202]. The second method is to perform RNA capping during transcription by adding a cap analog like ARCA (anti-reverse cap analog), which carries a methoxy group (–OCH_3_) in place of the 3′ hydroxyl group (–OH closer to m7G). When ARCA-capped mRNAs were compared to mRNAs capped by a conventional cap analog, the translation efficiency in rabbit reticulocyte lysates was twice as high [208]. Additionally, ARCA-capped mRNAs also have longer half-lives and initiate more protein expression in cultured cells [72, 275]. Recently, it was further shown that the co-transcriptional trimeric cap analog method of producing cap 1 structures can yield flexible capping and improved gene expression [88, 203],this technique was recommended for producing SARS-CoV-2 mRNA vaccines [183]. Regardless of the final application, properly capped mRNAs will be an essential feature of mRNA drugs.

### Poly (A) tail

The poly (A) tail also contributes to mRNA stability, and its length is positively correlated to translation efficiency [98, 155]. The poly (A) tail can be incorporated in the plasmid template, added via PCR, or added post-transcriptionally by enzymatic polyadenylation, which generates variable lengths of poly (A) tails. In mammalian cells, the poly (A) length is about 250 nt, but it is gradually reduced during an organism’s lifetime. For mRNA drugs, it has been shown that a poly (A) tail length of approximately 100 nt is optimal to minimize decay [192]. A segmented poly (A) approach of adding smaller spacer lengths between poly (A) segments in the DNA template can lead to higher translation efficiency and reduce plasmid recombination in *E. coli*, as compared to the use of conventional homogeneous poly (A) tails [221].

### Modified nucleotides

Codon composition is also important for mRNA translation. GC-rich mRNA sequences are associated with higher protein expression levels but not higher translation rates, suggesting that the GC-rich sequences are more efficiently transcribed [114]. Optimization of mRNAs by using a GC-rich sequence and incorporating 5-methylcytidine (m5C) and pseudouridine (Ψ) was found to minimize immunogenicity and increase translation efficiency [111, 245]. Recently, N1-methylpseudouridine (m1Ψ) has been used in mRNAs for SARS-CoV-2 mRNA vaccines, as these mRNAs elicit a less immunogenic response and have protein production more than one order of magnitude greater than Ψ-containing mRNAs [8, 156]. Importantly, m1Ψ does not produce miscoded peptides either during translation or during RNA duplex formation [107].

mRNAs encoding the same polypeptide but using different codons can produce dramatically different amounts of protein [206, 243]. Synonymous codon changes may affect protein conformation and stability, change sites of post-translational modification, and alter protein function [223]. It is therefore not surprising that synonymous mutations have been linked to numerous diseases [188, 199]. Thus, codon usage should be carefully considered and optimized during mRNA drug design since different codons may affect RNA and protein structures as well as the overall safety of the drug. Rare codons generally do not limit protein production in mammalian cells or bacteria [256]. However, fluorescent protein genes with synonymous codons produced proteins with fluorescent properties that differed due to protein folding [187]. Structural and functional studies should be conducted to test the possible effects of codon optimization. For example, mass spectrometry may be used to analyze cryptic peptide expression from constructs for mRNA drug application.

### Secondary structures

mRNAs contain 5′- and 3′-UTRs that form secondary structures important for correct ribosome scanning and dissociation, regulation of translation, and stability of the mRNA. Generally, 5′-UTR contains upstream open reading frames (uORFs) and stable secondary structures that regulate translation efficiency. Translation of uORFs may titrate translation initiation complexes, dissociate the ribosome from the mRNA following termination of the uORF, or downregulate uORF-containing mRNAs via nonsense-mediated decay (NMD) [99, 246]. For example, G4 structures in the 5′-UTR have been shown to act as translation repressors, while G4 structures in the 3′-UTR affect mRNA localization [205]. During mRNA design, the Kozak sequence (gcc)gccRccAUGG is generally placed after the 5′-UTR sequence to improve translation efficiency [149],sequences may be derived from genes such as globin, Hsp70, axon dynein heavy chain 2 (DNAH2), and hydroxysteroid dehydrogenase (3β-HSD) [33, 245]. To improve mRNA stability, a 3′-UTR may be modified from hemoglobin subunit α (HBA) and subunit β (HBB) genes [57] or albumin (ALB), heat-shock protein 70 (Hsp70), tyrosine hydroxylase (TH), or collagen alpha 1 (COL1A1) gene [102, 197]. Recently, many studies have begun to explore how UTRs affect mRNA translation efficiency, but the topic requires further investigation for application in mRNA drug design.

**Fig. 1:**
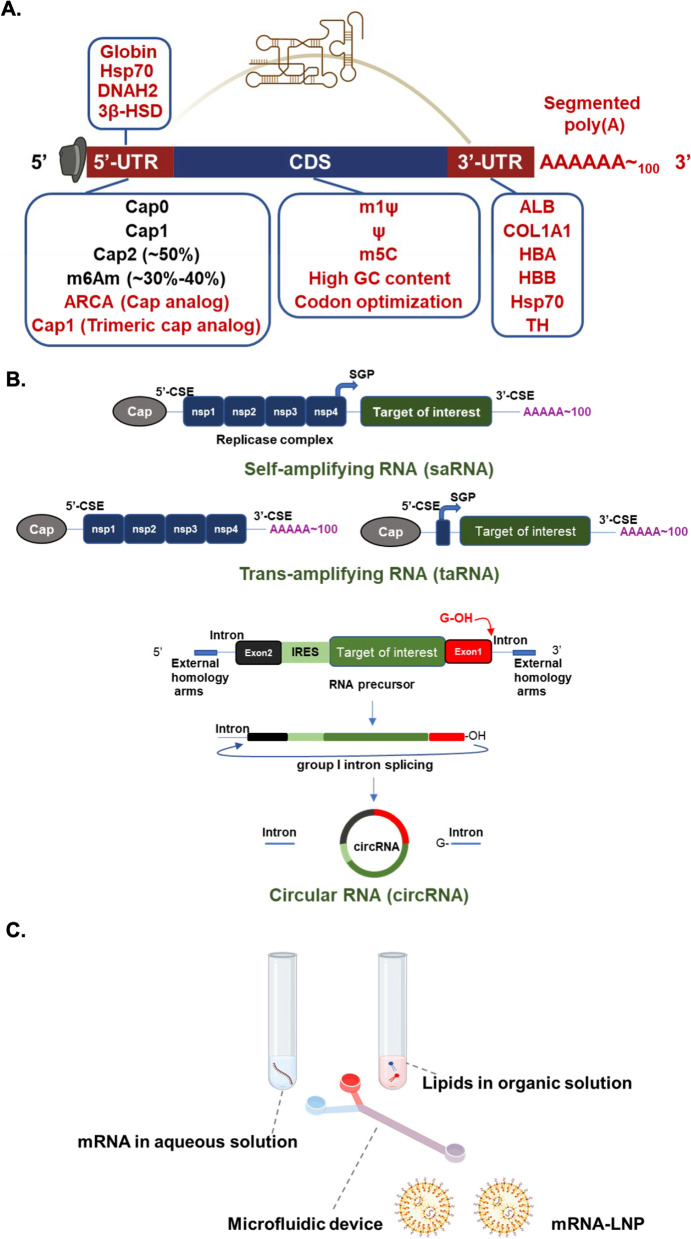
Types of synthetic mRNA for therapeutic application. A. Structural elements of mRNAs include the protein-encoding open reading frame (ORF), 5′ and 3′ untranslated regions (UTRs), 5′ cap structure, and 3′ poly (**A**) tail. mRNA drug design may involve several modifications to these structural elements in order to improve stability and protein expression. For example, the 5′-UTR and 3′-UTR from heat shock protein 70 (Hsp70) may be utilized, uridine can be replaced with m1Ψ, and optimized codons can be included to generate desirable higher-order structure and promote stable expression. Several possible mRNA modifications are shown in red. **B** In addition to conventional mRNAs, different synthetic RNA types include self-amplifying RNA (saRNA), trans-amplifying RNA (taRNA) and circular RNA (circRNA). saRNAs consist of two ORFs; One is np1-np4, which forms a replication complex, and the other is the target mRNA. saRNAs may be divided into a set of two taRNAs to avoid large size and low encapsulation efficiency. A circRNA with an internal ribosome entry site (IRES) linking a target of interest can be generated by using a self-splicing intron to circularize precursor mRNA. The construct can then be purified by HPLC. A permuted intron–exon (PIE) splicing strategy can allow for the fusion of exons with half-intron sequence and external homology sequence to enhance splicing efficiency [247]. After producing the precursor mRNA with IVT, GTP, and Mg^2+^ are added as cofactors to drive group I intron splicing,circularized mRNA typically exhibits a longer half-life than its counterpart linear mRNA. **C** Production of mRNA-LNPs (lipid nanoparticles). mRNA and lipid solutions should be dissolved in aqueous and organic solvents, respectively. The desired solution allows mRNA stability and facilitates the easy mixing of both solutions based on polarity. These components were then mixed using a microfluidic device to obtain stable and uniform mRNA-LNP nanoparticles

The secondary structure of the 5′-UTR affects protein production [239], but the impacts of coding sequences (CDS) and 3′-UTR secondary structures are not yet well understood. A recent study utilized different modified nucleotides to illustrate the relationship between protein production and mRNA secondary structure in different regions. The authors found that high protein expression could be correlated with increased secondary structure in the CDS and the 3′-UTR. They also found codon optimality and greater CDS secondary structure synergistically increased mRNA functional half-life [143]. Modifications of a conventional linear mRNA are summarized in Fig. 1A.

### Synthetic mRNA types

Many studies have been conducted with the aim of developing synthetic mRNAs with desirable characteristics, such as small-size, resistance to degradation, and high-yield of protein expression. There are three types of therapeutically applicable synthetic mRNAs, including non-replicating mRNAs (nrRNAs), self-amplifying mRNAs (saRNAs), and circular mRNAs (circRNAs) (Fig. 1B). Conventional synthetic linear mRNAs are a subtype of nrRNA with bases that have been modified to improve immunogenicity (as described in “Modified nucleotides”). In contrast, saRNAs have auto-replicative activities, so they only require lower doses than nrRNAs to achieve comparable protein expression levels. For example, 10 ng of saRNA is enough to induce immunogenicity toward SARS-CoV-2 in mice [140], and only 5 μg of saRNA is needed for clinical treatment [162]. Most saRNA designs and production strategies are based on positive-sense alphavirus genome, which consists of two ORFs, non-structural proteins (np1–np4 form the replication complex), and structural proteins including capsid and envelope proteins (E3-E2-6K-E1) [209]. To prepare saRNA constructs by IVT, the structural proteins are replaced with a target of interest and controlled by the virus subgenomic promoter (SGP). Within the 5′-and 3′-UTRs, one can find viral conserved sequence elements (CSEs), which are responsible for specific RNA amplification by alphavirus replicase [209] (Fig. 1B). Since the length of saRNAs is larger than 10 kb and encapsulation efficiencies of large mRNAs are low, saRNA formulations are relatively difficult to deliver. To overcome these barriers, an alternative approach is to divide the saRNA into two transcripts called trans-amplifying mRNAs. One is IVT-generated mRNA that encodes for alphavirus replicase, and the other is trans-replicon (TR) RNA encoding the target under the control of SGP. The short TR-RNA can then be amplified in trans by alpha replicase with suitable speed and efficiency [193]. So far, this approach has been used to generate a bivalent taRNA (trans-amplifying RNA) vaccine against chikungunya virus (CHIKV) and Ross River virus (RRV), which induces specific and potent humoral and cellular immune responses [193]. Thus, taRNA-based multivalent vaccines against infectious diseases may be achievable in the near future.

Unlike linear mRNAs, circRNAs are closed-ring molecules formed by covalent bonding, and this circular structure protects RNAs from exonuclease degradation. Thus, circRNAs have a median half-life of at least 2.5 times longer than linear mRNA isoforms in mammalian cells [132, 266]. An extensive set of circRNAs are known to be generated in eukaryotic cells by noncanonical RNA splicing events. These endogenous molecules have been shown to regulate a variety of physiological processes, either by acting as sponges or competitors for microRNAs and proteins, or by encoding functional peptides in response to stress [260]. Most endogenous circRNAs lack the essential elements for cap-dependent translation, but the molecules can be engineered by inserting an internal ribosome entry site (IRES) for protein expression. To produce circRNAs by IVT, half self-splicing introns can be fused with exons to direct the target RNA circularization (Fig. 1B),otherwise, the IVT-generated RNA can be directly circularized upon processing by T4 RNA ligase [17, 37, 247]. Notably, the intron splicing strategy has been used to create a SARS-CoV-2 vaccine (LNP-circRNA encoding the RBD region) with a potent cellular response and effective protection against different variants of concerns in mice and macaques. In addition, this vaccine also produces higher and more sustainable antigen production compared to LNPs with conventional linear base-modified mRNAs [168].

## Delivery systems for mRNA vaccines and drugs

A well-designed mRNA may have improved translation efficiency, but the delivery of naked mRNA is not a feasible approach since the cellular uptake efficiency of naked mRNA is extremely low [182]. One reason for this poor uptake is that negatively charged mRNA is repelled by the anionic cell membrane. Moreover, the typical size of mRNA drugs is much larger than other molecules that easily diffuse into the cell, and naked mRNA is also vulnerable to degradation by nucleases [77]. To overcome these obstacles, several approaches have been taken for delivering mRNA in vitro and in vivo. Different strategies for mRNA delivery are described in detail below.

### Lipids

Cationic lipids can quickly form complexes with negatively charged nucleic acids. Moreover, the hydrophilic and hydrophobic interactions between lipid polar head groups and nonpolar tails promote the formation of liposomes, which protect and efficiently deliver encapsulated cargo (e.g., mRNA) to target cells. Cationic lipids, including DOTAP (1,2‑dioleoyl‑3‑trimethylammonium-propane) and DOTMA (1,2‑di‑*O*‑octadecenyl‑3‑trimethylammonium-propane) can efficiently interact with negatively charged mRNA and have been used to deliver mRNA in vitro and in vivo [136, 171]. However, treatments with cationic lipids may be immunogenic and toxic, as they have been found to cause interferon-gamma secretion and liver damage [119, 133]. In addition, the positively charged lipids can react with negatively charged serum proteins, which may result in reduced efficacy or toxicity [131].

To avoid potential toxicity from cationic lipids and to increase delivery efficiency, researchers have begun to utilize ionizable lipids. These lipids are neutral at physiological pH but become positively charged at low pH, which allows for nucleic acid–lipid complexes to form in acidic buffer. After encountering the target cells, the complexes can fuse with the negatively charged endosomal membrane to cause lipid bilayer destabilization and mRNA cargo release into the cytoplasm [237]. Ionizable lipids can be further complexed with other lipid components to form ionizable LNPs that are well-suited for efficient mRNA delivery. For example, hydrophobic and rigid cholesterol can be included to fill gaps between lipids and promote LNP stability [39]. Moreover, helper lipid components like 1,2‑distearoyl-sn‑glycero‑3‑phosphocholine (DSPC) can be complexed in the LNP to enhance mRNA delivery by supporting fusion between cellular and endosomal membranes, which facilitates both cellular uptake and endosomal release [115]. Another potentially beneficial molecule type is PEG lipids, which contain a PEG moiety connected to alkyl chains that can be anchored into the membrane bilayer of LNPs. The inclusion of PEG lipids reduces opsonization by serum proteins, suppresses aggregation, and limits reticuloendothelial clearance [100, 124]. Generally, mRNAs and lipids are respectively dissolved in aqueous and organic solutions, and then these two components are mixed with a microfluidic device to produce mRNA-LNP complexes (Fig. 1C). Currently, several FDA-approved ionizable lipids have been applied to delivery of mRNA in clinical applications. For instance, the BNT/Pfizer vaccine Comirnaty contains ionizable lipid ALC-0315, while the Moderna vaccine Spikevax includes ionizable lipid SM-102. Both companies utilize a microfluidic system to make LNPs with SARS-CoV-2 spike mRNA for their mRNA vaccines [81].

The development of mRNA COVID-19 vaccines was greatly facilitated by the use of LNP vectors, which can deliver mRNA cargo to host cells and trigger an appropriate immune response [91]. LNP vectors have several key advantages over other delivery technologies. For instance, the LNPs are minimally immunogenic, able to carry multiple mRNAs in one formulation, amenable to use at multiple dosages, and easy to scale up. In spite of these advantages, there are disadvantages that need to be overcome, such as cytotoxicity of the lipid components (e.g., PEG-lipids) [78, 214]. Hence, it is important to further optimize LNP components and gain a deeper understanding of LNP uptake and the immune system in the effort to design more clinically effective LNP delivery systems. Meanwhile, other delivery systems such as polymers and peptides are also under systematic investigation.

### Polymers

Cationic polymers have been reported to condense negatively charged nucleic acids into polyplexes that can be shuttled across cell membrane, and this technology has great potential for improving delivery of mRNA-based therapeutics. Several studies have explored the use of different polymers in mRNA delivery. For example, polyethyleneimine (PEI) has been applied to deliver mRNA for HIV gag (the major structural polyprotein for HIV virus assembles), and this approach can induce HIV‑1 gag-specific immune responses in mice [272]. In another study, PEG-PAsp (DET) has been used to deliver brain-derived neurotrophic factor (BDNF) mRNA to nasal neurons, leading to repair of neurological architecture and function following intranasal administration [13]. Furthermore, local injection of PEG-PAsp (TET) nanomicelles loaded with mRNA of runt-related transcription factor (RUNX) 1 mRNA into knee joints could suppress the progression of osteoarthritis in mice [2]. However, the therapeutic application of most of the synthesized polymers consisting of high-molecular weight and branching designing leads to efficient gene delivery efficiency with large cytotoxicity issues [131]. Hence, researchers are working on designing biodegradable polymers using natural biopolymers or using surface modification techniques to reduce the cytotoxicity of synthesized polymers [11, 116, 122]. Moffett et al. have successfully demonstrated the delivery of mRNA into T cells by using a biodegradable poly (β-amino ester) (PBAE) polymer in mice model [144]. Recently, another group has modified a PBAE polymer to design the potent inhalable delivery of mRNA [178].

Cationic polymeric nanoparticles have several advantageous characteristics, such as simplicity of synthesis, ability to interact with mRNA in aqueous solution, long-term storage stability, and the ability to carry large nucleic acids. Despite substantial advances in this technology, polymeric nanoparticles have not been widely studied in clinical trials due to their high cytotoxicity and relatively low transfection efficiency [96, 158]. To improve the clinical applicability of polymer nanoparticles, precise development of biocompatible polymer nanoparticles with low batch-to-batch variability will be required.

### Peptides

Positively charged amino acids, such as lysine and arginine, have electrostatic interactions with the negatively charged backbones of nucleic acids, and these interactions can be exploited to improve mRNA delivery. Importantly, some cationic peptides can even complex with anionic mRNA molecules to form stable nanoparticles, which protect the mRNA from nuclease degradation. For example, protamine is an arginine-rich peptide that can be used to stabilize and deliver mRNA, and protamine-mRNA complexes have been applied in cancer and viral vaccines [63, 194]. However, the mRNAs in complex with protamine were found to be poorly translated, which may limit the potential for the development of protamine-based mRNA applications [268]. Alternatively, cell-penetrating peptides (CPPs) are molecules with membrane-penetrating capabilities, and some CPPs are rich in positively charged amino acids. These properties suggest that it may be feasible to use CPPs for mRNA delivery. Cationic amino acid-containing CPP HELP-4H, which was modified from bee venom melittin, has been used to deliver luciferase mRNA to the HCT116 colon cancer cell line and promote protein expression [3]. Meanwhile, the arginine-rich RALA peptide has been applied for OVA mRNA delivery, and this approach can induce antigen-specific CD8^+^ T cell immune responses in mice [226]. Iterative development of peptide-based mRNA delivery systems has helped to improve the synthesis process and increase the flexibility of peptide design. However, the tight binding of mRNA with peptides typically affects mRNA release and endosome escape ability, lowering the target protein expression level [109, 264]. Consequently, it will be important to design peptides with balanced positive charge to allow their escape from endosomes and improve the therapeutic applicability of peptide-based mRNA delivery technologies.

### Other delivery systems

In addition to those mentioned above, scientists have developed several other strategies to deliver naked mRNA to cells. One is electroporation, wherein an electrical field is applied to increase the permeability of the cell membrane to mRNA. Electroporation has been utilized to successfully deliver mRNA to human dendritic cells for tumor antigen loading and mouse zygotes for gene editing﻿ [84, 229]. Alternatively, the gene gun mRNA delivery method involves shooting mRNA-loaded metal particles into cells. This method has been used for stimulating protein expression in vitro and in vivo. For example, the gene gun approach has been applied in the development of a vaccine for tick-borne encephalitis virus [137, 167]. Microinjection is another method of delivering mRNA. In this method, a micropipette is used to inject membrane-impermeable mRNA into the intracellular space of living cells to induce protein expression [146]. Importantly, electroporation, gene gun, and microinjection are only suitable for local mRNA delivery. For systemic delivery, You et al. used extracellular vesicles (EVs) produced from human dermal fibroblasts to encapsulate collagen mRNA for collagen-replacement therapy. In contrast to mRNA-LNPs, treatment with mRNA-EVs did not induce inflammatory infiltration in local tissue, which suggests that EVs may be another viable approach for mRNA delivery [265].

## Routes of administration for mRNA vaccines and drugs

**Fig. 2:**
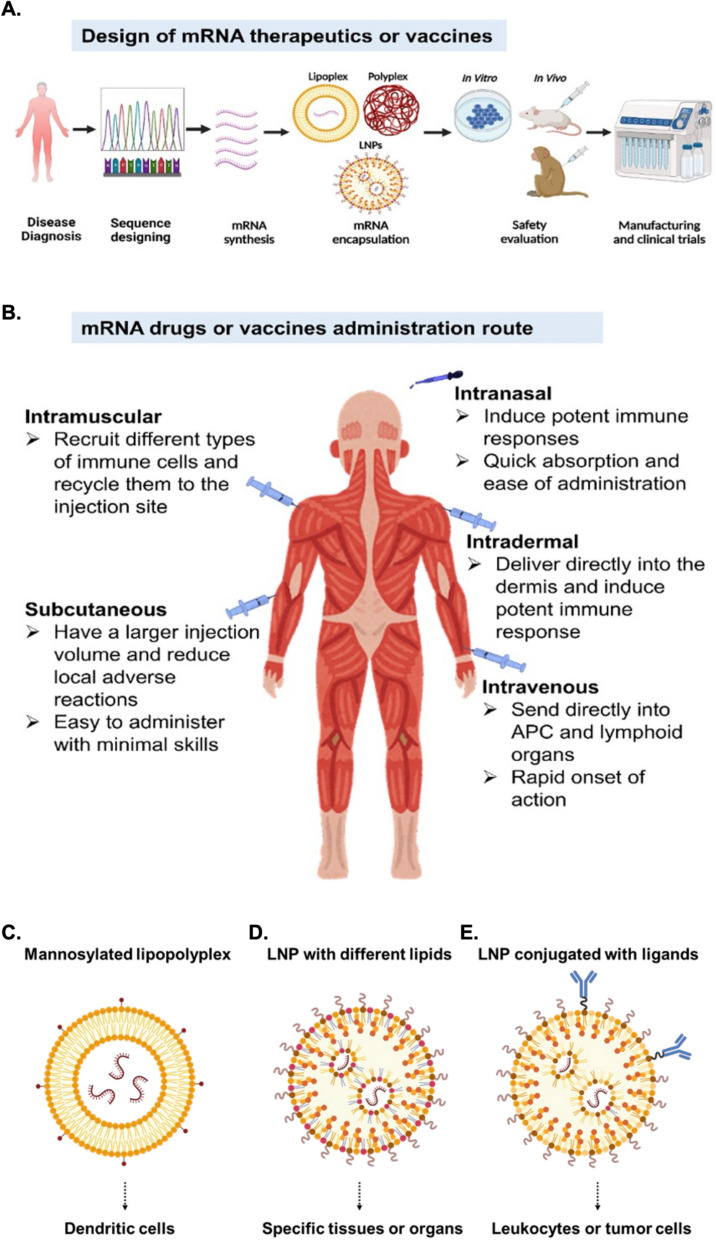
The potential for mRNA therapeutics and vaccines. **A** The process of creating novel mRNA drugs from sequence design to clinical translation. The first step is to design an mRNA sequence for a particular disease. Once mRNA is synthesized successfully, the delivery system should be established. Recently, lipid nanoparticles (LNPs) have been proven to be an efficient delivery tool. Animal models and cell-based assays may be used to evaluate the mRNA drug during preclinical testing. The mRNA drug can progress to clinical trials after successful pre-clinical tests. **B** The administration route is a key consideration when developing mRNA drugs for different diseases. The route might vary depending on the disorder and the type of drug. As an alternative to injections, nasal delivery is a promising method for treating infectious diseases and neurological disorders. Targeted delivery strategies for mRNA. mRNA drugs can be delivered to specific cells, tissues or organs. **C**–**E** The delivery of mRNA drugs to specific cells, tissues, or organs can be achieved using targeted mRNA delivery strategies. **C** Mannosylated lipopolyplexes can be delivered to splenic dendritic cells; **D** LNPs with different lipid components can be delivered to specific tissues or organs. For example, delivery using LNPs with shorter chains of ionizable lipids induced protein expression in liver, while LNPs with longer chains of ionizable lipids induced mRNA translation in spleen. Moreover, ionizable cationic, permanently cationic or zwitterionic helper lipids can be used for efficient mRNA expression in liver, lung or spleen. **E** LNPs conjugated with ligands can be used to delivered to leukocytes or tumor cells. For example, LNPs conjugated with antibody against CD5 can be delivered to T cells, while LNPs conjugated with antibody against CD117 can be delivered to hematopoietic stem cells

After successfully designing mRNA sequences and encapsulation strategies (Fig. 2A), it is essential to choose an appropriate route of administration to ensure sufficient amounts of mRNA are delivered to the target cells. Different routes of administration may be best suited for mRNA-based drugs depending on the disease and type of therapeutic mRNA. The administration route is known to robustly affect mRNA-mediated antigen expression levels and immune responses. For COVID-19, both FDA-approved mRNA vaccines are delivered via intramuscular (i.m.) injection; this route is the most preferred for the delivery of vaccines due to its marginally invasive nature and rapid absorption of drugs [19]. However, mRNA vaccines can stimulate antigen-specific immunity when delivered by several routes, including i.m., intravenous (i.v.), hypodermic (i.h.), intradermal (i.d.), intraperitoneal (i.p.), subcutaneous (s.c.), intranasal (i.n.), intranodal, and intrasplenic treatments, as demonstrated in Fig. 2B [28, 201, 269]. Currently, the mechanisms involved in antigen expression after delivery by different administration routes are being investigated. For instance, Huang et al. designed an LPX/RBD-mRNA complex and studied immunogenicity after delivery by five different administration routes (i.v., i.m., i.h., i.d., and i.p.). They found that mice immunized via i.v., i.m. and i.h. treatments showed similar levels of protein expression, and lower expression levels were seen in mice receiving i.d. and i.p. injections. Importantly, significant differences were found in the IgG subtype and cytokine responses when comparing between each of the different routes of administration [94]. In another study, Baharom et al. demonstrated that the administration route of vaccination can affect intratumoral myeloid cells. In contrast to s.c. injections, i.v. vaccination produced a larger number of tumor-specific CD8^+^ T cells [16].

A recent preprint by Künzli et al. suggests that systemic administration of mRNA drugs enhances both humoral and cell-mediated immunity. Moreover, the authors propose that when two different administration routes are combined (e.g., i.n. and i.m.), the number of resident memory T cells can be increased [118]. To illustrate how both nanoparticle type and administration route influence protein expression, one recent study compared different carriers encapsulating self-amplifying mRNA (saRNA). In particular, the study compared solid lipid nanoparticles (SLNs), polymeric nanoparticles (PNPs), corosolic acid (CA)-modified lipid nanoparticles (cLNPs), and ionizable lipid nanoparticles (iLNPs). All four nanoparticles were administered via i.m., i.d., and i.n routes. The study showed that iLNPs produce the highest IgG responses, followed by cLNPs and SLNs after i.m. and i.d. injections in BALB/c mice [6]. While the i.v. and i.d. routes yielded comparable antibody responses with i.m. injection, it is still unclear how each route of administration might affect RNA vaccine efficacy. As a result, medical trials are underway to evaluate various administration methods, but i.m. injections are still the most common route.

Mucosal pathogens contribute high rate of mortality and morbidity for infectious diseases. Therefore, mucosal immunity triggers the protection against pathogen infection and defense against most infectious diseases. In 2013, authors efficiently summarized the clinical trials of rotavirus vaccines and indicated a strong correlation between serum IgA and vaccine protection [157]. These findings are important since mucosal immunity may provide better protection against infection than humoral immunity. This reduces the entry of pathogens into the interior of the body as well as prevention of infection in the first place [120]. In support of this idea, higher level of nasal IgA against the influenza virus vaccine was shown to provide stronger protection than one with a lower IgA response. Tamura et al. summarizes the cross protective immunity against subtype-specific immunity and heterosubtypic immunity in mice recovered from influenza A infection. The major reason for this is the large amount of nasal IgA which can cross react with further viruses challenge as compared to IgG [212]. Physiologically, mucosal immunity contributes a primary role in preventing disease transmission, while serum IgG might serve mainly to prevent severe infectious diseases and have little effect on disease transmission. For COVID-19 vaccines, it is indispensable to prevent disease transmission by carriers. Recently Azzi et al. studied the specific immunity at the mucosal site from BNT162b2 vaccinated individuals. They found that the neutralizing antibody and IgG level is lower in saliva as compared to serum. This might be attributed that the immunization route plays a major role to activate the mucosal immunity. Therefore, immunization of booster dose via nasal or oral route might further enhance the mucosal immunity and limit the viral dose from the entry route [12]. To support this hypothesis Tang et al. showed that combination of i.m and i.n route not only enhanced the SARS-CoV-2 immunity but also induce protection against emerging variants [213]. Recently, an intranasal or intraoral vaccine was found to regulate mucosal immunity to fight SARS-CoV-2 severe infection. Since the precise role of mucosal immunity is still unclear in terms of SARS-CoV-2 transmission, however, this helps to recruit local innate immunity and induced memory T cells [79]. Further deep research may be helpful to analyze the uncovered relationship between disease transmission, mucosal immunity, and mRNA vaccines.

The ability to deliver an mRNA to a specific organ or cell type would help to address many different medical needs. As such, commercial demand has driven work to explore how targeted mRNA delivery methods could be applied to direct therapeutic mRNA medicines to specific cell types. For example, Perche et al. showed that mannosylated lipopolyplex can target splenic dendritic cells more efficiently than control lipopolyplex [159]. Similarly, Kim et al. found that LNPs with ionizable lipids 241C10 to 246C10 can efficiently target liver sinusoidal endothelial cells (LSECs) [108]. Moreover, Liu et al. observed that ionizable lipids with different lengths of hydrocarbon tails or helper lipids with different charges can be used to guide organ-selective mRNA-LNP distributions [130]. Chen et al. also applied the lipid 113-O12B to formulate LNPs that can specifically target lymph nodes [36]. Researchers also observed that tuning the content of PEG lipids in LNPs causes mRNA-loaded LNPs to target different types of liver cells in vivo. The same study offered evidence that mRNA-LNPs with mannose-PEG lipid specifically target liver sinusoidal endothelial cells [108]. Additionally, it has been reported that encapsulating additional molecules in LNPs can direct the complexes to different tissues. For example, inclusion of DODAP, 18PA or DOTAP causes the LNPs to respectively target liver, spleen or lung [38, 50]. Moreover, Veiga et al. intravenously injected leukocyte-targeted IL-10 mRNA encapsulated in an anti-Ly6c-antibody-decorated LNP into a mouse model of inflammatory bowel disease (IBD). This treatment increased the expression of IL-10 in colon, consequently reducing intestinal inflammation and preventing colitis pathogenesis [233]. Using a similar LNP design, Rosenblum et al. intraperitoneally injected anti-EGFR-antibody-decorated LNPs encapsulating Cas9 mRNA and PLK1 single guide RNA (sgRNA) to disseminated ovarian-tumor-bearing mice. They found that the LNPs were efficiently taken up by ovarian tumors, which led to gene editing of the PLK1 locus in tumor cells. As a result, tumor growth was inhibited and survival of the mice was extended [176]. A recent study reported the successful delivery of a target gene to the heart using a CD5 antibody-conjugated to LNP-mRNA. Gene delivery efficiency increased from 7 to 83% when using the CD5-LNP-mRNA [180]. Furthermore, Breda et al. applied LNPs conjugated with antibody against CD117 to deliver genome-editing RNAs or pro-apoptotic mRNAs to hematopoietic stem cells in vivo. The genome-editing LNP led to effective correction of hematopoietic sickle cells, while delivery of pro-apoptotic factors could be used to condition patients for hematopoietic stem cell transplantation [26]. Such modification strategies provide a means of guiding mRNA-based medicines to specific target cells or organs. Current strategies for mRNA targeted delivery are summarized in Fig. 2C–E.

## Applications of mRNA vaccines and drugs

**Fig. 3:**
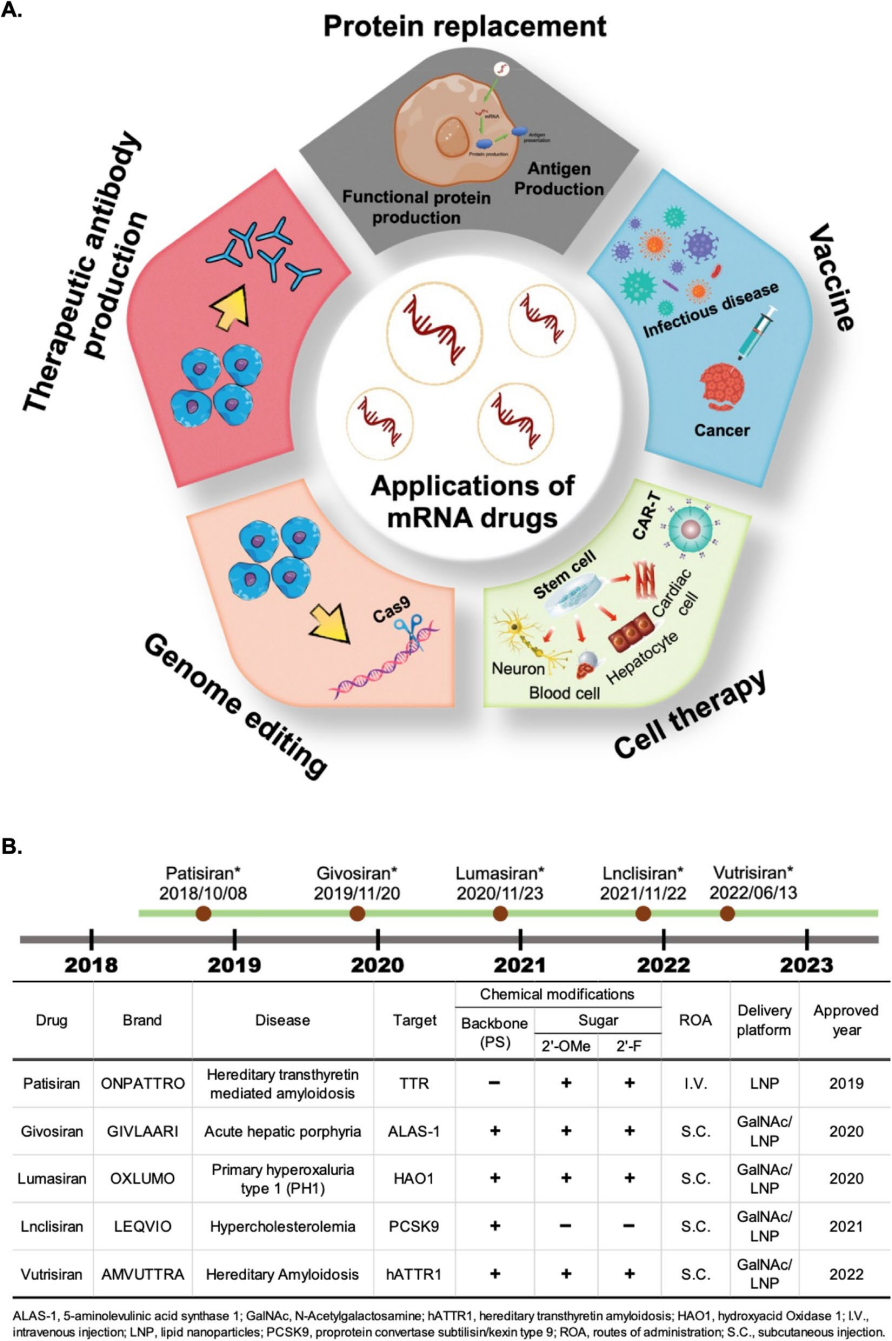
Medical applications of RNA drugs and FDA approved RNAi drugs. **A** The applications of mRNA-based drugs for disease therapy include vaccines, cell therapy, therapeutic protein production, and protein replacement. mRNA-based drugs have proven to be a potent competitor in vaccine development. Along with prevention of infectious diseases, mRNA vaccines may also be used in the treatment of cancer. Regarding cell therapies, mRNAs can be applied in CAR-T cell therapy, or treatments may also be developed to target disease-relevant cell types, such as cardiac cells, blood cells, hepatocytes and neurons. For therapeutic protein production, mRNAs can be translated into patient’s own cells to produce therapeutically active proteins. These protein-encoding mRNAs can be used for antigen presentation, functional protein expression, or Cas9 protein expression for target gene modification. Furthermore, small RNAs (e.g., siRNA or miRNA) may be useful to inhibit overactive genes. For protein replacement, protein-coding mRNAs can be used as gain-of-function therapies, replacing non-functional mutant proteins to restore normal physiological function. **B** The table shows U.S. FDA-approved RNAi drugs currently in clinical use

mRNA-based therapy is expected to be used for a variety of diseases that are refractory to current treatments, such as infectious diseases, metabolic genetic diseases, cancer, cardiovascular disease, cerebrovascular diseases, and others [164]. mRNA drugs can offer further advantages of high efficiency with low side effects, and ease of production. As such, mRNA vaccines have already proven to be a safe and effective strategy for limiting the spread of COVID-19 [34]. The first mRNA vaccine to receive emergency use authorization was made by BNT/Pfizer (BNT162b2), and its approval was quickly followed by approval of the Moderna vaccine (mRNA-1273). These vaccines were each ~ 90% effective in terms of preventing wild-type SARS-CoV-2 infection in fully vaccinated individuals and ~ 80% effective in partially vaccinated adults [74, 161, 216]. Several strategies have been found to improve the efficiency of COVID-19 mRNA vaccines, such as mutating two proline codons to stabilize the S protein translation product or using modified mRNA encoding prefusion S protein (BNT162b2 and mRNA-1273). In addition, a growing number of mRNA-based drugs are under development for clinical therapeutic applications and the approach has even been applied in drug development efforts for immune cell-related diseases. Despite the rapid progress that has been made in the field, the use of mRNA-based therapeutics for many diseases such as AIDS and cancer will require further research and development. In this section, we summarize current progress in development of mRNA-based drugs and their applications in disease treatment. The schematic diagram is shown in Fig. 3A. There are currently five FDA-approved RNAi drugs in clinical use, namely Patisiran (2019), Givosirna (2020), Lumasiran (2020), Lnclisirna (2021), and Vutrisirna (2022). The chemical modifications and delivery methods of these drugs are shown in Fig. 3B.

### Vaccines for infectious diseases

Vaccination is a well-known and widely applied means of preventing a large number of diseases. The successful deployment of numerous vaccines has prevented or helped to cure many life-threatening infections. To date, several different types of vaccines have been approved for clinical use, including inactivated or attenuated pathogens, subunits, and viral vectors. For most vaccines, development from preclinical research to clinical trials has taken around 15–40 years. The fastest vaccine developed prior to SARS-CoV-2 was against mumps and comprised an attenuated virus. The recent breakthrough in mRNA vaccines has drastically altered the expected vaccine development timeline, as it proved possible to develop a vaccine for emergency approval in only a few months [117]. Recent studies on mRNA vaccines have focused on evaluating the protection efficacy of many infectious diseases, including influenza virus, respiratory syncytial virus (RSV), Zika virus (ZIKV), rabies virus, Ebola virus, *T. gondii*, and *Streptococcus* spp., as well as new COVID-19 variants (Tables 1, 2). Notably, the key role of mucosal immunity in defending against infectious diseases has also garnered attention, which led to the development of novel vaccine delivery systems [95, 241]. Figure 2A demonstrated the sequence designing for the clinical translation process of mRNA-based drugs or vaccines.

**Table 2:** Regulatory agency-approved mRNA vaccines

Brand (Generic) name	Drug name	Originator company	Approval year	Regulatory agency	Disease	Delivery platform	Route	Target-based actions
Comirnaty (Tozinameran)	BNT-162b2	BioNTech SE	2020	EMA	SARS-COV-2	LNP	i.m. injection	COVID19 spike glycoprotein modulator
			2020	HC				
			2020	MHRA				
			2021	FDA				
Comirnaty Original/Omicron BA.4–5 (Tozinameran/Famtozinameran)	BNT-162b2 bivalent (WT/OMI BA.4/BA.5)		2022	EMA	SARS-COV-2	LNP	i.m. injection	COVID19 spike glycoprotein modulator
			2022	HC				
			2022	MHRA				
Spikevax (Elasomeran)	mRNA-1273	Moderna	2020	HC	SARS-COV-2	LNP	i.m. injection	COVID19 spike glycoprotein modulator
			2021	EMA				
			2021	MHRA				
			2022	FDA				
Spikevax Bivalent Original/Omicron BA.1 (Elasomeran/Imelasomeran)	mRNA-1273.214		2022	EMA	SARS-COV-2	LNP	i.m. injection	COVID19 spike glycoprotein modulator
			2022	MHRA				
Spikevax Bivalent Original/Omicron BA.4–5 (Elasomeran/Davesomeran)	mRNA-1273 containing SARS-CoV-2 omicron-specific bivalent (BA.4/BA.5)		2022	EMA	SARS-COV-2	LNP	i.m. injection	COVID19 spike glycoprotein modulator
			2023	SMC				

*EMA* European Medicines Agency, European Union, *FDA* Food and Drug Administration, United States, *MHRA* Medicines and Healthcare products Regulatory Agency, United Kingdom, *HC* Health Canada, Canada, *SMC* Swissmedic, Switzerland, *LNP* Lipid nanoparticles

#### COVID-19

As of March 30 2023, many mRNA vaccine candidates for COVID-19 are in preclinical trials and 43 are under evaluation in clinical trials. Of these 43, seven have advanced to phase 3 trials [250]. Much of this work involves the evaluation of new strategies to create mRNA vaccines. Unlike non-replicative mRNA-based drugs, which are FDA-approved, new classes of mRNA drugs, such as self-amplifying mRNA (saRNA) or circular mRNA (cRNA), are being studied in preclinical and clinical settings and hold great promise. For instance, saRNA vaccines may be able to overcome the need for multiple doses and require much lower doses than currently licensed mRNA vaccines [162]. Other vaccines have been designed to overcome the continual decline of vaccine efficacy against new emerging variants. In this regard, different strategies such as the administration of booster doses or bivalent vaccines are expected to improve future vaccine efficacies [248].

#### Influenza

An influenza pandemic shook the world in 1918, and the virus has long been recognized as a leading cause of death. The influenza virus can be sub-categorized into four types (A, B, C, and D), three of which are known to infect humans [227]. Influenza A and B are the most common causes of seasonal epidemics, while influenza C typically causes mild disease in humans. The first attenuated influenza A vaccine was developed after a decade of virus outbreaks [18], and according to the World Health Organization (WHO), the vaccine has significantly reduced the mortality rate. Currently, several different types of vaccines have been approved for influenza, including inactivated whole virus, inactivated split, live attenuated, inactivated subunit, and recombinant vaccines. However, almost every year, the genetic makeup of the virus slightly changes and efficacies of available vaccines against seasonal influenza strains wane [31]. In 2012, a research team from CureVac GmbH (Tübingen, Germany) demonstrated the potential use of an mRNA vaccine against the influenza virus. In their work, they designed an mRNA for vaccination the against influenza A virus and showed protection efficacy in mice, ferrets, and domestic pigs. They also suggested that designing mRNA vaccines against the seasonal flu would be more amenable to scale-up in a short time period and could overcome waning vaccine efficacies [160]. Later in 2013, scientists from Novartis Vaccines and Diagnostics designed mRNA vaccines against H7N9 within 8 days of a major outbreak of the virus [87]. Unfortunately, progress in clinical studies was stalled because of low mRNA stability, a suboptimal delivery system, and lack of GMP facilities and protocols. Since then, several approaches have been taken to produce mRNA vaccines for influenza that are capable of protecting from different strains of the virus and providing a long-term immunity.

Importantly, co-infection of SARS-CoV-2 and influenza increases the risks of mortality and morbidity, and some studies support the hypothesis that influenza infection can facilitate SARS-CoV-2 infection. In light of the problems posed by co-infection, researchers are now making major efforts to design combination vaccines that can protect from both viruses in one shot [86, 262]. Hence, combined mRNA vaccines are expected to become available for mitigating the risks of deadly viruses and future pandemics.

#### Flaviviruses

Viruses of the Flaviviridae family include ZIKV, Japanese encephalitis virus, yellow fever virus, and Dengue virus (DENV) [253]. In 2015 and 2016, ZIKV outbreaks caused a global health crisis, especially owing to its association with fetal death [169]. Infections of this virus are by a single serotype, so it should be relatively straightforward to design vaccines without having to account for different strains [154]. Unfortunately, the vaccines against ZIKV can show cross-neutralization of DENV, as the envelope proteins of the two viruses share approximately 50% similarity. Therefore, poorly designed Zika vaccines can induce low levels of neutralizing antibodies against DENV serotypes. These antibodies can be problematic, as they can enhance the effects of subsequent infections with different DENV serotypes, leading the infected individual to experience severe symptoms [47]. Only a few researchers have succeeded in developing vaccines that can protect against ZIKV and DENV-2 serotype infection [53]. Due to problems with antibody dependent enhancement (ADE) of Dengue fever, no Zika vaccine is currently licensed and only one DENV vaccine (Dengyaxia) has been approved by the US Food and Drug Administration [58]. Recently, Qdenga (TAK-003) was approved for dengue prevention by the Indonesian and Brazilian governments [211]. There is also one Zika DNA vaccine (VRC5283) that has completed phase I trials and is now under evaluation in phase II clinical trial (NCT03110770), and two Dengue DNA vaccines are currently in phase I trials (NCT00290147 and NCT01502358). Meanwhile, one mRNA vaccine against Zika infection is in phase I studies (prM-E antigen,NCT03014089 and NCT04064905), but there is no mRNA vaccine against DENV infection that has yet to enter clinical trials. The major limitation of DENV vaccine design is providing robust efficacy against all four serotypes. Although the four DENV serotypes share a largely conserved amino acid sequence in the envelope protein, major differences exist in a key binding loop. These differences impede vaccine efficacy for some serotypes and allow their escape from neutralizing antibodies induced by vaccination, which increases the risk of ADE [1, 244].

### Personalized cancer vaccines

Cancer immunotherapies activate the immune system to inhibit tumor growth and may even be able to eliminate cancer from the body [173]. One type of immunotherapy, cancer vaccines, is intended to introduce tumor-specific antigens or tumor-associated antigens to antigen-presenting cells (APCs) in order to boost immune responses and activate T cells that kill cancer [189]. The first cancer vaccine was approved in 2010 by the US FDA. This vaccine involved replacement of GM-CSF-activated APCs in the patient and extended life expectancy by 4.1 months, according to the phase III clinical trial results [29]. To optimize cancer vaccine efficacy, several approaches have been taken, including codelivery of cancer antigens with immune stimulatory molecules, promotion of immune-activating conditions in the tumor microenvironment, and combination of vaccines with traditional medical treatments like chemotherapy or radiotherapy. In addition, mRNA vaccines are being considered for the treatment of cancer, as it is highly desirable for next-generation cancer medications.

**Fig. 4:**
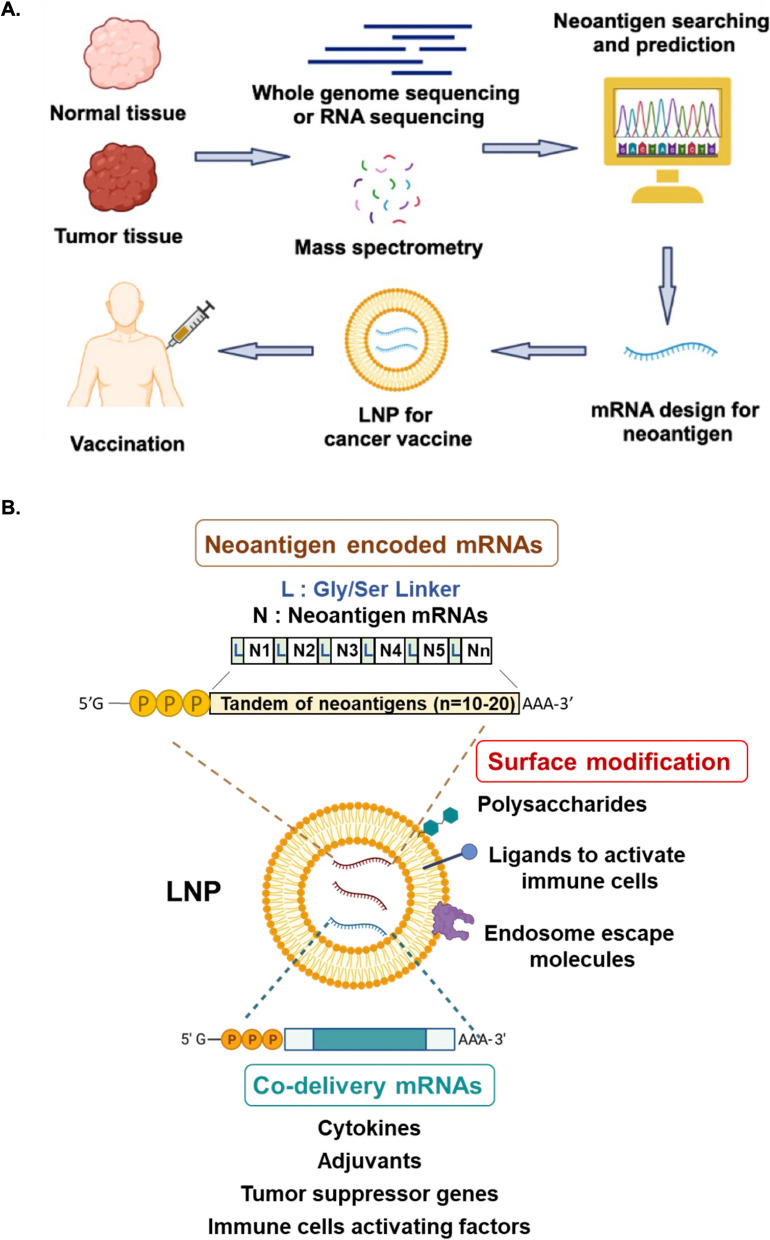
Development and modification strategies for mRNA-LNP cancer vaccines. **A** Neoantigens can be identified and validated by whole genome sequencing, RNA sequencing or protein expression from normal and tumor tissues. Validated neoantigens can be utilized for the design of mRNA therapeutics, which may be delivered using LNPs. **B** The different neoantigen mRNAs could be linked tandemly to be synthesized and incorporated into LNPs for delivery as a personalized cancer vaccine. Co-stimulatory molecules, such as IL-12 and IL-27, may be co-delivered to activate immune cells. Other co-stimulatory molecules could include tumor suppressor genes like PTEN and p53 to induce cancer death, adjuvants like STING^V155M^ and glycolipid to activate CD8^+^ cells or invariant Natural Killer T (iNKT) cells, or macrophage polarization factors like IRF5 and IKKβ to induce M1 cell polarization. Surface modifications can be made to the LNPs, such as the addition of polysaccharides to induce immune response or the inclusion of endosome escape molecules to enhance mRNA release into the cytosol for expression

Tumor antigen could be divided into tumor-associated antigen (TAA) and tumor-specific antigen (TSA) or neoantigen. TAA could be expressed in tumor and normal tissues, but it is an abnormally higher expression in tumors and lower in normal tissues. Due to TAA being a non-mutated self-antigen, poor T-cell responses will be observed in clinical immunotherapy [123]. Neoantigen might offer an ideal targeting antigen designed for a personal cancer vaccine. Neoantigens are produced by genomic nutation in tumors and unique antigen will be translated by wrong RNA splicing and unexpected post-translational protein modification [54]. Advanced techniques like next-generation sequencing (NGS) or mass spectrometry could be applied to differentiate these neoantigens by comparing with their whole-genome and mRNA sequencing or dysregulated protein from normal and tumor tissues. Possible major histocompatibility complex (MHC) binding epitope candidates could be predicted by some algorithms to identify neoantigen mRNA for cancer vaccine application [172, 258] (Fig. 4A).

For most cancer vaccines, dendritic cells (DCs) serve as key targets of antigens and adjuvants, as these are the major APCs used to prime T cell immunity. Successful delivery of mRNAs to DCs and macrophages in lymphoid tissues can be accomplished using one RNA-lipoplexes (RNA-LPX) by simply adjusting the net charge without changing the well-characterized composition or adding surface ligands [113]. An mRNA-LPX vaccine has shown potency after in situ vaccination,the vaccine delivered IL-12 mRNA to transform the tumor microenvironment and reprogram DCs to prime T cells [89]. In another approach, DC vaccines can be generated ex vivo by engineering RNA-LPX with iron oxide nanoparticles (IONPs) before infusion back into the patient. The use of RNA-IONPs had benefits such as enhanced DCs transfection efficiency and easy tracking of DCs migration by magnetic resonance imaging (MRI). Most importantly, injection of the RNA-IONP-treated DCs led to superior inhibition of tumor growth [71]. Another mRNA delivery approach that has been explored in the context of cancer vaccines is bacteria-derived outer membrane vesicles (OMV). Genetic engineering was performed on the RNA binding protein L7Ae and lysosomal escape protein listeriolysin O (OMV-LL) integrated on the surface of the OMV, which allows OMV-LL to adsorb box C/D sequence-labeled mRNA antigens through L7Ae binding. This complex could deliver mRNA to DCs in vivo, which was followed by endosome escape and cross-presentation of the antigen. Treatment with this new delivery platform induced obvious therapeutic effects in an animal model of colon cancer [126].

In another study, an injectable hydrogel was formed with graphene oxide (GO) and polyethyleneimine (PEI). This hydrogel was infused with mRNA antigen and an adjuvant (R848, a palmitic acid-modified TLR7/8 agonist), and it persisted for at least 30 days after subcutaneous injection for targeting skin dLN-DCs. Such a long-lasting exposure allowed for robust generation of specific antibodies and antigen-specific CD8^+^ T cells, and the vaccine could inhibit tumor growth after only a single treatment [263]. Similarly, an ovalbumin-encoding mRNA and R848 adjuvant coated with a lipid-polyethylene glycol (lipid-PEG) shell could effectively induce the adaptive immune response and cause the expansion of OVA-specific CD8^+^ T cells in mice [97]. The stimulator of interferon genes protein (STING) signaling is important for type I IFN in the innate immune system and has also been applied as an adjuvant for cancer vaccines. mRNA-encoding constitutively active STING^V155M^ was effective at inducing CD8^+^ T cells with a ratio of 5:1 of antigen/adjuvant. Furthermore, vaccination with LNP-antigen mRNA-STING^V155M^ mRNA caused significant regression of HPV + TC-1 tumors and prolonged survival time in mice [224]. In another strategy, sugar capsules composed of mannose and carrying mRNA could efficiently activate DCs and promote antigen presentation, stimulating immune cells to recognize polysaccharides of bacteria and respond to pathogen-associated molecule patterns (PAMPs) [204]. Using another platform called mRNA Galsomes, researchers co-delivered nucleoside-modified antigen-encoding mRNA, glycolipid, and a ligand α-galactosylceramide (α-GC) to dendritic cells for activating invariant natural killer T cells (iNKTs) and CD8^+^ T-cells. Vaccination with mRNA galsomes enhanced the responsiveness to treatment with a PD-L1 inhibitor in B16-OVA melanoma and enhanced the infiltration of cytotoxic T lymphocytes, natural killer cells, and iNKTs to eliminate tumor cells in mice [234]. In another study, immunosuppression was reversed in the tumor microenvironment by applying an excess of positive LNPs carrying untargeted tumor RNA to prime the peripheral and intratumoral environment for response to immunotherapy, with systemic and intratumoral myeloid cells co-expressing PD-L1 and CD86. The addition of immune checkpoint inhibitors to activate PD-1^+^ CD8^+^ cells synergistically boosted anti-tumor activity [191], and local radiotherapy also synergistically promoted the cancer vaccine anti-tumor activity by enhancing DC sensing of tumor antigens [20, 185, 186]. In addition, a simple strategy to replace protein adjuvants is under development, with investigators adding short-double strand RNA (dsRNA) to LNPs as an adjuvant that can activate the innate immune receptor RIG-I and increase the effectiveness of cancer vaccination [219].

Several studies have used LNP delivery tools to modify the tumor microenvironment and promote an immune-active state, which is at least partially determined by cytokine profiles. A single dose of intratumor with IL-12 mRNA delivered by LNP to mice could induce IFNγ and CD8^+^ T-cell dependent tumor regression [89]. IL-12 mRNA drives TH1 transformation in the tumor microenvironment, and MEDI1191 (a human IL-12 mRNA) is in a phase I trial (NCT03946800). In addition, intertumoral delivery of IL-12 and IL-27 mRNAs could synergistically induce strong infiltration of immune effector cells into murine B16F10-derived melanoma tumors, representing a new strategy for cancer treatment [127]. Besides affecting cytokines to modulate the tumor microenvironment, nanoparticles carrying mRNAs for interferon regulatory 5 (IRF5) and its activating kinase IKKβ were shown to induce M1 polarization of immunosuppressive tumor-associated macrophages and promote tumor regression [270]. IL-27 stimulates multiple lineages of immune cells, and IL-27-induced C–C motif ligand 5 (CCL5) contributes to IL-27 mediated anti-tumor activity. As such, intratumor delivery of CCL5 mRNA with LNPs was shown to significantly reduce tumor growth, and IL-27 was found to induce robust CCL5 production by T cells resulting in antitumor activity [93]. Other co-stimulatory molecules OX-40L/CD80/CD86 could be delivered by LNPs and activate APCs and T cells, which produced an immune-active state in the tumor microenvironment [75]. Interestingly, some suppressor genes like PTEN and p53 are also important for tumor microenvironment-induced cancer cell death. PTEN mRNA-LNPs reversed the immunosuppressive nature of the tumor microenvironment by promoting CD8^+^ T cell infiltration and enhancing the expression of proinflammatory cytokines including IL-12, tumor necrosis factor-α (TNF-α), and IFN-γ. These cytokines reduced suppressor cells such as regulatory T cells and myeloid-derived suppressor cells [128].

Polymer/lipid hybrid NPs with targeting peptide CTCE and carrying p53 mRNAs (CTCE-p53 NPs) can actively target CXCR4 chemokine receptors that are expressed in hepatocellular carcinoma. Intravenously administered CTCE-p53 NPs combined with anti-PD1 mAb treatment caused significant regression of established RIL-175 tumors by restoring P53 in HCC and reversing the immunosuppressive tumor microenvironment [257]. Another tool with great promise for cancer therapy is in situ T cell transfection. Using this approach, CD3-specific antibodies incorporated in LNPs (aCD3-LNPs) were shown to transduce and temporarily activate 2–7% of circulating T cells and 2–4% of splenic T cells, which had strong anticancer effects [105]. Recently, there has been a major breakthrough in the treatment of pancreatic cancer using mRNA-based personalized cancer vaccines. Pancreatic ductal adenocarcinoma (PDAC) ranks as the seventh leading cause of cancer deaths in the world [210]. PDAC patients are largely insensitive to immune checkpoint inhibitors and exhibit high recurrence rates with a 5-year survival of only 8–10% after surgery [179, 261]. Recently, Rojas et al. developed a new personalized cancer vaccine for PDAC composed of 10–20 neoantigen mRNAs. The vaccine is delivered using an LNP and pretreatment with Atezolizumab to boost T cell immunity. They addressed chemotherapy treatment is not affecting the effect of cancer vaccine for delaying PDAC recurrence [175]. A summary of the studies on mRNA cancer vaccines described above is provided in Fig. 4B.

### mRNA-enhanced cell therapy

**Fig. 5:**
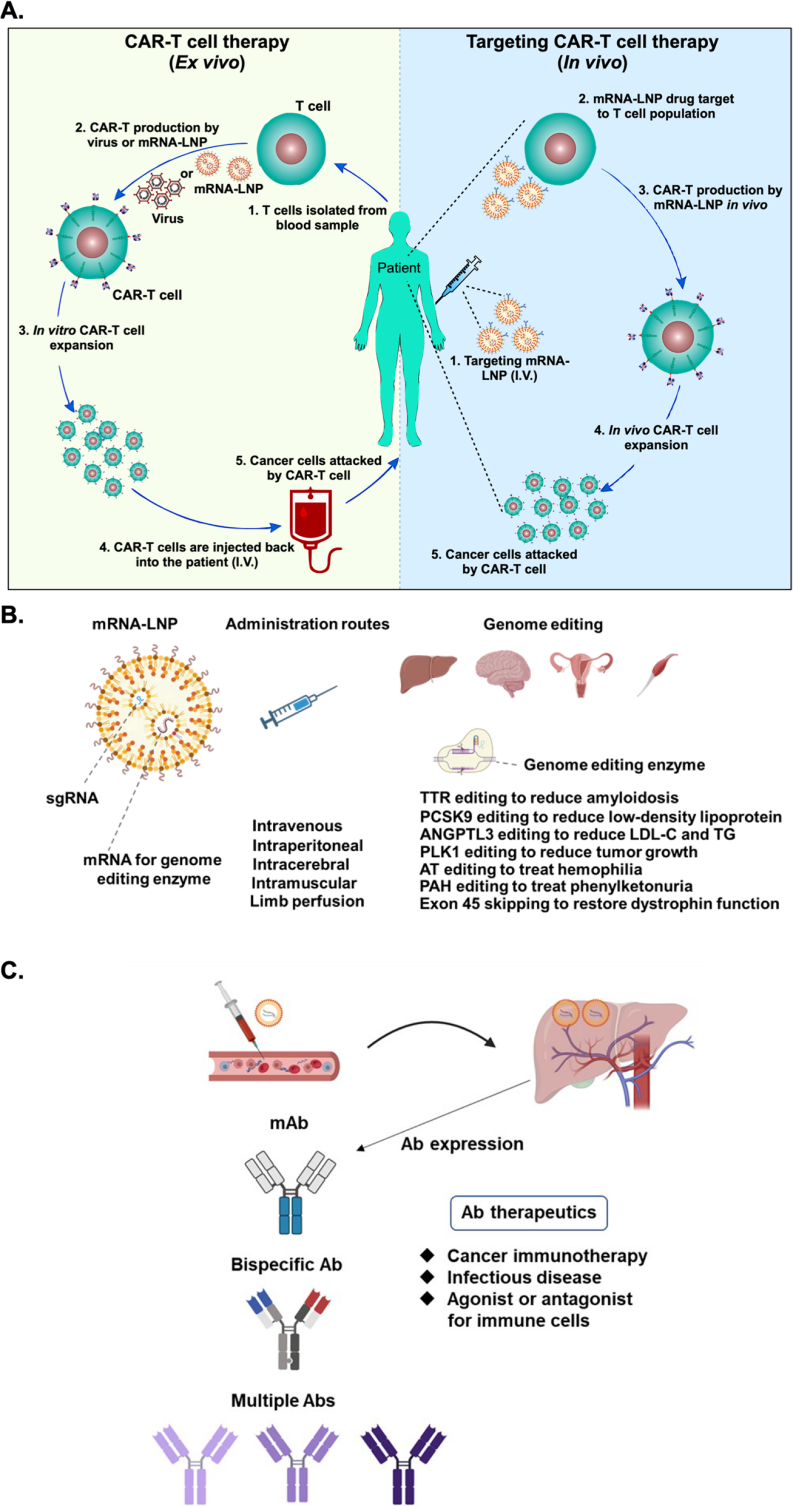
mRNA-based new modalities for disease treatments. **A** Current CAR-T technology requires the isolation of T cells from a patient and processing of the isolated cells into CAR-T cells (right panel). Next-generation CAR-T therapy is expected to be more effective, shorten the therapeutic timeframe and lower the cost. CAR-T cells may be generated in patients through intravenous injection of targeted mRNA-LNPs (left). **B** LNP-encapsulated mRNAs encoding genome editing enzymes and other components may be administrated through different routes. Genes of transthyretin (TTR), proprotein convertase subtilisin/kexin type 9 (PCSK9), angiopoietin-like 3 (ANGPTL3), polo-like kinase 1 (PLK1), antithrombin (AT), phenylalanine hydroxylase (PAH) or exon 45 was edited and eventually alleviated the disease progression. sgRNA: single guide RNA; LDL-C: low-density lipoprotein cholesterol; TG: triglycerides (TG). **C** Utilization of mRNA drugs for engineering therapeutic antibodies (Abs). LNP-based delivery can be applied to generate different types of therapeutic Abs at higher levels and with more sustainable expression than conventional antigen injections. This approach could be applied to a variety of diseases, including cancers and infectious diseases

Cell therapy is one of the most promising new areas of medicine, and mRNA technologies may be key to realizing its potential [76]. In many ex vivo cell therapies, target proteins can be modified by mRNA treatments in vitro, and then the mRNA-modified cells may be injected into the patient to cure disease. Currently, there are several mRNA-based cell therapies in clinical trials, including TriMix-based immunotherapy (ECI-006), autologous cell therapy CAR-T MCY-M11 (MaxCyte), and Cartesian therapy [5]. In addition, Zhong et al. reported the use of chemically modified mRNA encoding TGF-β3 (TGF-β3 cmRNA) to enhance the therapeutic efficacy of bone marrow stem cells for repair of cartilage defects [267]. Such mRNA-based therapies, including CAR-T cell therapy (Fig. 5), have great potential for clinical use in the prevention and treatment of a wide variety of diseases. It is hoped that this advanced technical platform can partially replace traditional drugs as a new treatment frontier with novel methods [106].

### Therapeutic genome editing

The use of LNPs to deliver nucleic acid components has been explored in the context of therapeutic genome editing. Compared to viral-based gene delivery methods, mRNA-LNP-based genome editing methods are more transient and have less potential for adverse effects, such as nuclease-induced off-target mutagenesis or viral vector-mediated immunogenicity [170]. Most applications of mRNA-LNP-mediated genome editing are still in preclinical development, but one has progressed to clinical trials. This approach has largely been explored in the context of modifying expression of transthyretin (TTR) or proprotein convertase subtilisin/kexin type 9 (PCSK9). For example, Conway et al. utilized LNPs comprised of a proprietary ionizable lipid to intravenously deliver engineered zinc finger nuclease for specific genome editing. Using this technique, the expression of TTR or PCSK9 could be successfully reduced in mice after treatment [41]. In another method, Liu et al. encapsulated Cas9 endonuclease mRNA and sgRNA targeting PCSK9 in bio-reducible lipid BAMEA-O16B-based LNPs. After intravenously administering the LNPs, serum levels of PCSK9 in mice were significantly reduced [129]. In addition to TTR and PCSK9, other proteins have been targeted with similar approaches. For instance, Rosenblum et al. applied novel amino-ionizable lipid L8-composed LNPs to intracerebrally deliver Cas9 endonuclease mRNA and sgRNA targeting polo-like kinase 1 (PLK1), a kinase necessary for mitosis, in aggressive orthotopic glioblastoma in vivo. This treatment inhibited tumor growth and improved mouse survival [176]. Qiu et al. also applied tail-branched bioreducible lipidoid 306-O12B-composed LNPs to encapsulate and intravenously deliver Cas9 endonuclease mRNA and sgRNA targeting angiopoietin-like 3 (Angptl3), an enzyme that regulates the level of plasma lipoprotein. Administration of these LNPs significantly reduced serum angptl3 protein, as well as low density lipoprotein cholesterol and triglyceride levels. Interestingly, the therapeutic effect of this genome editing method lasted for at least one hundred days after a single treatment [166]. Kenjo et al. treated humanized Duchenne muscular dystrophy (DMD) mice by intramuscular injection or limb perfusion with ionizable lipid TCL053-based LNPs encapsulating Cas9 mRNA and sgRNA targeting the dystrophin gene. As a result of the treatment, exon 45 skipping was induced, and the expression of dystrophin protein was restored in the mice. These effects were coincident with a reduction of damaged and regenerating myofibers after the treatment [104]. Moreover, Han et al. intravenously treated a mouse model of hemophilia with ionizable lipid 246C10-based LNPs encapsulating Cas9 mRNA and sgRNA targeting the antithrombin gene. The treatment reduced expression of antithrombin and increased expression of thrombin, leading to less severe bleeding-associated phenotypes in the mice [80]. Moreover, Brooks et al. treated phenylketonuria (PKU) mice with an ionizable lipid SM-102-based LNP encapsulating adenine base editors (ABEs) mRNA and sgRNA targeting pathogenic variants of the phenylalanine hydroxylase (PAH) gene by retro-orbital injection. This treatment led to editing of the liver pathogenic PAH gene and recovery of blood phenylalanine level in the mice [27]. In yet another application, Rothgangl et al. applied novel ionizable-based LNPs to intravenously deliver ABE-encoding mRNA and sgRNA targeting PCSK9 to the livers of mice and macaques. After treatment with the LNPs, editing of the targeted gene locus was confirmed. Moreover, the level of plasma PCSK9 was reduced and consequently blood low-density lipoprotein was lowered as well [177]. Importantly, recent clinical trial results show that the serum TTR protein concentrations decrease in patients after the treatment with NTLA-2001, which consists of LNPs derived from proprietary lipid LP01 and encapsulated Cas9 mRNA and human TTR gene-targeting sgRNA. Although the clinical trial is still ongoing, the successful development of a product to this point supports the idea that in vivo mRNA-LNP-based genome editing may be a viable therapeutic strategy [60, 67]. The application of mRNA-LNP techniques in therapeutic genome editing is summarized in Fig. 5B.

### Protein replacement therapy

Protein replacement therapies can be used to treat diseases caused by deficiencies or mutations of certain proteins. This form of therapy is widely applied to treat blood disorders, lysosomal storage disease, and metabolic disorders [44]. Recently, researchers have explored the use of mRNA-based products in applications such as cancer treatment (described above) or the treatment of metabolic disorders by delivery of protein-encoding mRNAs. In this context, mRNA technology allows proteins of interest to be expressed in vivo for extended times, thereby overcoming challenges of delivering protein drugs that may be large, have low stability, or have high costs of production [232]. In one groundbreaking project, researchers first evaluated the delivery of a therapeutic protein to the myocardium in order to regulate the cardiomyocyte cell cycle [21, 85, 145]. However, direct delivery of a protein with intracellular action is difficult due to the very low levels of achievable protein delivery and the need for repeated injection. In contrast, delivery of mRNA can be used to generate high intracellular protein levels, and delivery may be accomplished without major immunogenic consequences. So far, there have been several reports supporting the idea that modified mRNAs could be useful tools for protein replacement therapies. For instance, one group optimized human VEGF-A mRNA delivery into the left ventricular region in swine and showed the treatment reduces myocardial fibrosis. Of note, i.v. and i.m. administration of the mRNA to rats and monkeys did not induce innate immune responses [32]. Since it is possible that the need for invasive administration routes for mRNA delivery might decrease interest in regenerative therapies, AstraZeneca recently designed an mRNA-LNP formulation that may be suitable for subcutaneous (s.c.) administration. In this formulation, steroid prodrug is added, which dramatically increases the level of protein production and promotes long-term expression [46]. Studies such as this promise to make mRNA drugs easier to administer in the future.

### Antibody therapy

Engineered therapeutic antibodies with one or multiple targets could be useful to boost the anti-cancer activities of endogenous or treatment-associated T or NK cells. For example, a trispecific antibody against HER2, CD3, and CD28 was shown to inhibit breast cancer growth in a humanized mouse model via a mechanism involving CD4-dependent inhibition of tumor cell cycle progression [198]. While this approach is promising, the half-life of antibodies in serum is limited. Therefore, LNP-based delivery of antibody-encoding mRNAs may be a feasible means of delivering antibody therapies. Most bispecific antibodies are limited in use due to concerns with manufacturing and long-term stability during storage. Furthermore, the serum half-life of bi-(ScFv)_2_ protein is less than 2 h, making continuous infusion a requirement for treatment [65]. To overcome this issue, an mRNA encoding bi-(ScFv)_2_ for CD3 x CLDN6 was formulated with a polymer and lipid-based transfection agent for i.v. administration and expression in the liver. The translated antibody levels reached a peak 6 h after treatment and were sustained for several days. This treatment could inhibit subcutaneous xenografts of CLDN6-expressing ES-2 ovarian carcinoma cells. Furthermore, delivery of another mRNA encoding bi-(ScFv)_2_ for EpCAM x CD3 (to target EpCAM on OV-90, an ovarian epithelial tumor cell line) also showed excellent activity and suggested that bispecific antibody-encoding mRNAs may be a robust means of treating cancer [207]. LNPs-mRNA technology has been applied for expressing therapeutic antibodies, including those for the treatment of HER2-positive breast cancer [181], anti-human CD20 mAb (rituximab) for the treatment of non-Hodgkin’s lymphoma [217], and anti-PD-1 mAbs for the treatment of intestinal cancer [255]. Another bi-specific antibody, XA-1, was designed to target PD-L1 and PD1. This antibody could completely block the PD1/PD-L1 pathway to prevent intestinal cancer [254]. Regarding infectious diseases, there are more than 250 million carriers of Hepatitis B virus (HBV) in the world, and these individuals have elevated risk of developing other liver diseases. Treatment with antibodies against HBV surface antigen (HBsAg) is an effective means of reducing these risks, but it is a major challenge to maintain therapeutic levels of antibodies long term. For this purpose, an mRNA drug encoding three anti-HBsAg antibodies was developed and found to reduce serum HBsAg levels in treated mice after one booster dose [35]. Similarly, an LNP-encapsulated mRNA encoding one human monoclonal antibody against chikungunya virus was i.v. administered to mice before virus infection and protected from lethality, diminished signs of arthritis, and reduced viremia to an undetectable level 2 days after inoculation [112]. Based on the studies described above, mRNAs encoding therapeutic antibodies may be especially applicable for treating cancer and infectious diseases. As an alternative to i.v. infusion of LNP-encapsulated mRNA for therapeutic Abs, i.m. administration of an alphavirus replicon encoding ZIKV-117-neutralizing mAb by nanostructured lipid carrier (NLC) was shown to induce robust protection against Zika virus in mice [55]. Other delivery methods are also being explored, such as the nebulization of polymer-formulated mAb-encoding mRNAs to prevent SARS-CoV-2 infection [230]. Recently, three different designs of humanized EpCAM-CD3 bispecific-antibodies have been engineered, including EpCAM-CD3 CrossMab (knob-in-hole), EpCAM scFv-CD3 scFv (BITE), and EpCAM scFv-CD3 scFv-human Fc. When used with mRNA-LNP technology, these antibodies showed high specificity for killing EpCAM^+^ T cells. The EpCAM scFv-CD3 scFv-human Fc also significantly blocked OVCAR-5 xenograft tumor growth in vivo [69]. The applications of in vivo therapeutic antibody expression as treatments for specific diseases are summarized in Fig. 5C.

## Current landscape of mRNA-based drug pipeline

**Fig. 6:**
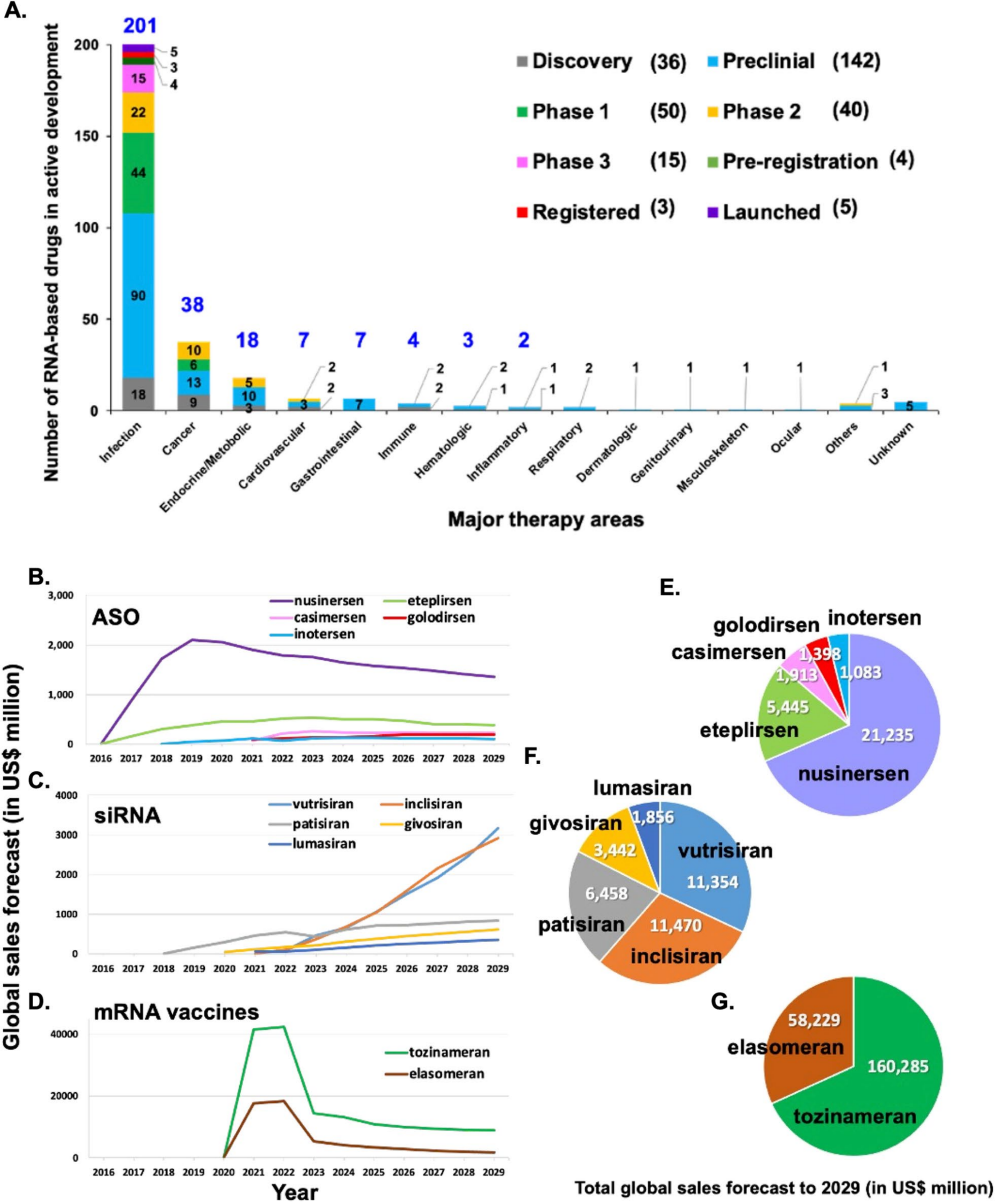
Development status and global sales forecast for mRNA-based drugs. **A** Composite development status of 316 mRNA-based medicines (excluding technologies that were discontinued, suspended or not updated for an extended period); analyzed with Clarivate’s Cortellis Competitive Intelligence Database on July 28, 2023. **B**–**D** Global sales forecasts to 2029 are based on analyst consensus, acquired from GlobalData’s Intelligence Center Database on June 26, 2023. **B** Five FDA-approved ASO (anti-sense oligo) drugs. **C** Five FDA-approved siRNA drugs. **D** Two FDA-approved mRNA vaccines are available on the market. Summary information regarding these drugs is provided in "Regulatory agency-approved drugs". Regulatory agency-approved drugs. **E**–**G** Total global sales forecasts (US$ million) for each drug up to 2029

The landscape of biomedical uses for mRNA is continuously and rapidly evolving. mRNA-based medicines in active development status (excluding those that had been suspended, discontinued, or development status not been updated for a long time) are searched and analyzed in Clarivate’s Cortellis Competitive Intelligence Database. Among the 316 active mRNA medicine records shown in database as of July 28, 2023, 57% are in discovery and preclinical stage (180 drugs), 39% entered clinical stages (125 drugs), 1.9% (6 drugs) have acquired (pre-)registration, and 1.6% (5 drugs) have been approved. mRNA medicines developed for infectious diseases occupied the largest portion (62.3%). Those targeting cancers were second most common (19.9%), followed by endocrine/metabolic (4.1%), immune (1.9%), respiratory (1.9%), cardiovascular (1.6%), gastrointestinal (1.3%), genitourinary (0.6%), inflammatory (0.6%), musculoskeletal diseases (0.6%), etc. (Fig. 6A). As compared to mRNA-based drugs, there are 408 active RNA interfering/modulating (including antisense oligonucleotides, siRNA, miRNA, etc.) RNA drug records, of which 75% are in discovery and preclinical development, 23% are in clinical stages, 1 drug acquired pre-registration, and 10 drugs approved. A list of approved mRNA and RNA modulating drugs are shown in Table 2.

### Drugs in clinical trials

The mRNA vaccines developed by Moderna and BNT/Pfizer during the COVID-19 pandemic provided novel weapons to combat disease and also helped to accelerate research and clinical trials on mRNA platforms [96].

In recent years, the world has witnessed a surge in the development and introduction of mRNA-based COVID-19 vaccines. These vaccines offer a promising solution to the challenges posed by mRNA stability and immunogenicity, which have been major roadblocks in the past. Researchers and scientists are optimistic that these new mRNA vaccines will provide effective protection and treatment for individuals affected by the virus. Improvements to the new generation of COVID-19 vaccines are expected to provide safer, broader, long-term protection and to induce cross-neutralization antibody responses against VOCs, such as Delta and Omicron variants [82, 83]. The introduction of bivalent vaccines overcomes the low neutralizing titer of existing vaccines against VOCs by targeting multiple strains (NCT05907044).

The development of mRNA vaccines is not limited to COVID-19. Work on mRNA vaccines targeted to many other infectious diseases is also in progress. Examples include vaccines for EBV (NCT05164094), RSV (NCT05127434), Zika (NCT04917861), and others [70]. Another example is the mRNA-1647 vaccine against cytomegalovirus (NCT05683457, NCT05085366; encoding cytomegalovirus pentamer complex and glycoprotein B antigens against cytomegalovirus), which is currently being tested in phase II/III trials. Moderna also has a seasonal qIRV influenza vaccine (mRNA-1010; against WHO-proposed strains) in phase III trials (NCT04956575), which makes this the fourth mRNA vaccine from Moderna to enter into phase III. Moderna is also investigating an mRNA vaccine (mRNA-1073) for combined protection from COVID-19 and influenza [31]. Another infectious disease that is being targeted with an mRNA vaccine is the Nipah virus. This virus causes a zoonotic disease, as most cases are transmitted via animals. However, person-to-person transmission can also occur and may lead to coma or death. Currently, there is no licensed vaccine or treatment for Nipah virus infection. Thus, NIH has launched an early-stage clinical trial evaluating an investigational vaccine to prevent infection with the Nipah virus (NCT05398796). Apart from targeting infectious diseases, mRNA vaccines against different types of cancer are now being thoroughly studied in clinical trials. For instance, an mRNA vaccine for advanced melanoma is in a phase II trial (mRNA-4157,NCT03897881). Phase I trial (NCT04161755) [175] is investigating surgical procedures followed by the administration of personalized tumor vaccines and PD-L1 inhibitors to delay the recurrence of pancreatic cancer in patients.

As mentioned previously, the unparalleled success of mRNA COVID-19 vaccines has stimulated research into the broader and deeper potential applications of mRNA-based protein expression. These new drugs may allow us to fight against diseases that are currently considered difficult to treat or untreatable. Currently, there are also a few ongoing clinical studies evaluating mRNA-LNPs for protein replacement therapies. A Phase I clinical trial for ARCT-810 (NCT04442347), which is a drug candidate for OTC deficiency, is completed in healthy adults. This drug is now being evaluated in Phase I/II study that is currently recruiting [236]. The use of mRNA drugs in cancer treatment has also shown significant improvements. The design of personalized vaccines or therapeutics targeting tumor-specific antigens such as claudin 18.2, claudin 6, and CD7 are revolutionizing cancer therapy and improving treatment outcomes.

Clinical trials are also underway to test CRISPR-modified primary human T cells for a first-in-class treatment of metastatic gastrointestinal cancer (NCT04426669). This treatment is expected to work without sacrificing cell viability or function, allowing for inhibition of a heretofore undruggable intracellular checkpoint cytokine-inducible SH2 containing protein (CISH) [4]. A clinical trial has also been initiated to treat sickle cell disease using the adenine base editor (ABE) (NCT05456880) [165].

Table 1 summarizes the registered clinical trials conducted in the US between December 2020 and June 2023 on mRNA-based drugs. These trials include treatments for COVID-19, cancer, infectious diseases, protein replacement, and gene editing. The data were obtained from ClinicalTrials.gov.

### Regulatory agency-approved drugs

The first-ever evidence of mRNA-based drug effectiveness against influenza was obtained in 1993 using mouse models [139]. Since then, many approaches have been developed to overcome the limitations of mRNA drugs. To improve this class of molecules, researchers have generally sought to limit degradation and increase stability of exogenous mRNA, enhance protein translation efficiency, and develop suitable mRNA delivery systems [45]. During the COVID-19 pandemic, the advantages of this technology allowed for extremely fast production of mRNA vaccines, while other types of vaccines were being developed at a much slower pace. Nevertheless, it is important to note that the first two mRNA drugs were approved by the US FDA after approximately three decades of technological development.

The first FDA-approved mRNA COVID-19 vaccine was BNT162b2, developed by BioNTech and Pfizer. The collaboration between these companies was initiated in 2018 in an effort to develop mRNA-based influenza vaccines [183]. At the time of the COVID-19 emergency, the group accelerated their efforts and produced striking clinical results only a few months after the SARS-CoV-2 sequence was decoded. In their efforts, the research team prepared two different mRNAs, called BNT162b1 and BNT162b2. BNT162b1 encodes only the receptor binding domain (RBD) of spike protein, while BNT162b2 encodes the full-length spike protein. Both mRNAs were encapsulated into LNPs, and their efficacies were tested in a randomized sample of 195 healthy participants. The preliminary data showed that BNT162b1 recipients reported high fever and severe local pain, so the safety and immunogenicity results only allowed BNT162b2 to progress into further clinical trials [238]. The United Kingdom was the first country to approve BNT162b2 on 2 December 2020 [121]. The high efficacy and safety of BNT162b2 convinced the WHO to grant approval for emergency use on 31 December 2020. As of 3 July 2022, BNT/Pfizer is estimated to have delivered more than 3.6 billion vaccine doses to around 180 countries and territories [274], and the global market was valued at approximately US$8.4 billion by 2022 [68].

Five mRNA vaccines (listed in Table 2) and 10 RNA modulating RNA drugs have been approved by regulatory agencies in the US and other countries as of June 2023 (listed in Table 3).

**Table 3:** Regulatory agency-approved RNA modulating RNA drugs

Drug type	Generic name	Brand name	Originator company	Approval year	Regulatory agency	Disease	Route	Target-based actions
ASO	Eteplirsen	Exondys 51	Sarepta Therapeutics	2016	FDA	Duchenne muscular dystrophy (DMD)	i.v. injection	DMD gene modulator
ASO	Nusinersen	Spinraza	Ionis Pharmaceuticals and Biogen	2016	FDA	Spinal muscular atrophy	Intrathecal injection	Survival motor neuron-2 (SMN2) gene modulator
ASO	Inotersen	Tegsedi	Ionis Pharmaceuticals	2018	FDA	Hereditary transthyretin mediated amyloidosis	s.c. injection	Transthyretin (TTR) gene inhibitor
siRNA	Patisiran	Onpattro	Alnylam Pharmaceuticals	2018	FDA	Amyloidosis, familial amyloid neuropathy, lipotoxic cardiomyopathy	i.v. injection	Amyloid protein deposition inhibitor, TTR gene inhibitor
siRNA	Givosiran	Givlaari	Alnylam Pharmaceuticals	2019	FDA	Acute intermittent porphyria, hepatic porphyria	s.c. injection	5-Aminolevulinate synthase 1 inhibitor
ASO	Golodirsen	Vyondys 53	Sarepta Therapeutics	2019	FDA	DMD	i.v. injection	DMD gene modulator
siRNA	Lumasiran	Oxlumo	Alnylam Pharmaceuticals	2020	FDA	Hyperoxaluria	s.c. injection	Hydroxyacid oxidase 1 modulator
ASO	Casimersen	Amondys 45	Sarepta Therapeutics	2021	FDA	DMD	i.v. injection	DMD gene modulator
siRNA	Inclisiran	Leqvio	Novartis	2021	FDA	Primary hypercholesterolemia	s.c. injection	Proprotein convertase subtilisin-kexin type 9 (PCSK9) inhibitor
siRNA	Vutrisiran	Amvuttra	Alnylam Pharmaceuticals	2022	FDA	Familial amyloid neuropathy, Stargardt disease	s.c. injection	TTR gene inhibitor

*ASO* antisense oligonucleotide, *siRNA* small interfering RNA, *FDA* Food and Drug Administration, United States, *i.v.* intravenous, *s.c.* subcutaneous injection

### Intellectual property landscape for mRNA drugs

The number of patent publications relating to mRNA vaccines began to increase in about 1995 and has grown exponentially starting from about 2009 [25]. According to searches on the Derwent Innovation platform (https://clarivate.com/products/derwent-innovation/), the statistics show more than 9600 related patents were registered by the end of 2021 [125]. Moreover, there are already over 15,000 patents valid and applied in major countries (such US, JP, EP, etc.) as end of June 2023. Major claims include optimization of methods on mRNA self-amplification, sequence or codon optimization, nucleotide modifications, cap or poly(A) tail modifications, and delivery by carriers, especially different LNP compositions. Among this patent filing activity, many delivery methods and cap/poly(A) tail modifications have been introduced in the recent few years [138] with mRNA delivery being of predominant interest [9]. The patent landscape of mRNA vaccines and technologies has been analyzed by several groups and comprises a complicated network of licensing, sublicensing, and partnerships (i.e., joint development and patent applications by multiple institutes and companies). Key players in the commercial space include companies, such as Moderna, BioNTech, CureVac, Arcturus, Arbutus, Acuitas, among others. Academic/research institutes, such as University of Pennsylvania, University of British Columbia, US National Institutes of Health (NIH), have also led the way as inventors of three key technologies used in producing approved COVID-19 mRNA vaccines, respectively nucleoside modification of synthetic mRNA, novel lipid components for mRNA delivery, optimization of SARS-COV-2 spike protein sequence [30, 66]. Patent litigations among these players started in 2021, with many alleging Moderna and/or Pfizer/BioNTech had infringed on LNP-related patents and seeking reasonable royalty compensation rather than an injunction [10, 249]. While researchers and developers of mRNA technology should pay attention to the outcomes of these litigations, reflection, and discussion about a more efficient mechanism of privatization of knowledge is needed. Many of the technologies were generated through extensive taxpayer-funded public research, as was the case for COVID-19 mRNA vaccines. Therefore, it is preferable to establish systems that can prevent the use of taxpayer money to financially benefit only a few companies rather than the society at large [61].

## Considerations and limitations of mRNA drugs

According to the central dogma, mRNAs can be designed to express any protein via ribosome machineries, so the technology can be used to treat many diseases. In addition, the production cost of mRNA is much lower than that of recombinant protein drugs [273]. However, mRNA-based drugs still have drawbacks that need to be overcome, such as immunogenicity, inefficient protein expression, and difficulties of large-scale production for clinical application. Therefore, further innovations in sequence design, LNP delivery systems, and manufacturing process optimization will be essential to promote the development of mRNA drugs [150]. Although the optimization of coding and non-coding mRNA sequences would be helpful to improve the translation efficiency [148, 215], the process of sequence designing requires workers to develop specific expertise. The immune system can recognize unmodified single-stranded RNA, which can reduce protein expression and lead to the development of reactogenicity [251]. To improve translation efficiency, modified nucleotides such as the commonly used m1Ψ may be incorporated into the mRNA [174]. Another challenge for mRNA-based drugs is that mRNA is a negatively charged molecule, so it cannot easily penetrate the lipid bilayer at the cell surface [190]. Furthermore, mRNA is vulnerable to phagocytosis by immune cells followed by degradation by nucleases. Intracellular release after uptake into target cells can pose another major challenge. In light of these issues, efficient and safe delivery systems are crucial for mRNA drugs. Currently, LNPs are the most clinically advanced mRNA delivery vehicle, and this well-developed technology is highly mutable and patentable [59]. At the same time, it is also necessary to optimize the design of synthetic mRNAs to solve problems associated with toxicity, aggregation, and leakage that may be associated with LNP-mediated delivery. Also, creating a uniform mRNA particle size can improve the stability of LNP delivery systems [151]. The purity of mRNA has a major impact on the therapeutic effect and safety of an mRNA drug. Therefore, it is also imperative to develop efficient purification systems for the synthesized mRNAs. Further improvements to mRNA-based drugs are still necessary and are major topics of ongoing research. For instance, it has been suggested that mRNA circularization may improve resistance to cellular ribonucleases, and novel biomaterials with targeting abilities could potentially improve biocompatibility, specificity and transfection efficiency. Overall, great improvements are expected in the near future for technologies to mediate in vivo delivery and to regulate durable protein expression.

## Conclusions and prospects of mRNA drugs

The development and authorization of mRNA vaccines for SARS-CoV-2 within one year of the beginning of the COVID-19 pandemic revealed the enormous potential of mRNA technology. Since then, the use of mRNA vaccines and drugs has remained under the spotlight, as the COVID-19 mRNA vaccines displayed superior efficacy and safety compared to other inactivated virus-, recombinant protein-, and viral vector-based vaccines. In addition, the mRNA vaccines benefitted from an extraordinarily short design-to-manufacture time (as short as 66 days) [42], which is in sharp contrast to the traditional vaccine development timeline of 10–15 years. With the major hurdles of poor stability and delivery into cells being finally solved by the incorporation of modified nucleotides [103] and cationic liposomes [136], mRNA is now considered one of the most powerful and widely applicable tools for prevention and treatment of disease. The insights gained from the process of advancing mRNA vaccines through all stages of pharmaceutical drug development to a commercial product will now be valuable for further efforts to advances novel mRNA applications in cancer vaccines and immunotherapy.

The topic of mRNA-based therapies is a state-of-the-art and rapidly evolving research field. The clear advantages of mRNA-based drugs over other biomedicines have attracted more and more industrial and academic researchers to undertake projects in this field. The merits of mRNA are numerous and include: (1) much shorter, simpler, and cheaper development and manufacturing processes than traditional biologics, which require complicated biological systems such as cell lines or *E. coli*; (2) little danger of pathogenic infection posed by in vitro enzymatic mRNA synthesis; (3) ability to modularize and switch manufacture to any specific protein by simply changing the mRNA sequence; (4) lack of mRNA integration into the genome, providing better safety profile than DNA-based therapeutics; (5) ability to target intracellular proteins that are once considered undruggable by antibody and protein drugs; (6) widespread potential applications, such as vaccines for infectious diseases (especially emerging diseases like COVID-19), cancer vaccine and immunotherapy, cell therapy, protein replacement therapy, and gene editing, among others; (7) potential for direct intervention in genetic diseases by hindering the expression of specific genes (pathological proteins) or introduction of proteins to compensate for deficiencies of functional proteins; (8) cellular and humoral immune responses induced by mRNA vaccines are widespread and effective, resulting in higher protection rates than conventional vaccines, as evidenced by the COVID-19 mRNA vaccines of Moderna [14] and BNT/Pfizer [7, 161]. With expected advancements and proper design of biomaterials, mRNA-based vaccines and drugs will almost certainly be in high demand for many clinical uses in the coming years.

Currently, more than 190 companies and institutes are engaged in the development of more than 310 mRNA vaccines and therapeutics. The developmental progress on these medicines’ ranges from discovery and preclinical studies to various stages of clinical trials. Among the products, 125 are in the clinical pipeline worldwide, with vaccines accounting for 65% and therapeutics 35%. Except for mRNA COVID-19 vaccines, most of these products are still in early clinical testing stages (Cortellis Competitive Intelligence Database as of March 5, 2023). Diseases that can be treated or prevented by mRNA drugs are typically regarded as limitless, and it is anticipated that mRNA will become the dominant platform for prophylactic vaccines within the next 15 years, as they comprise the largest proportion of vaccines in the pipeline and are expected to have a high success rate [259]. Along with SARS-CoV-2, other infectious diseases targeted by mRNA vaccines in development include seasonal influenza virus (NCT04956575, NCT05415462), human immunodeficiency virus type 1 (HIV-1) (NCT05414786), RSV (NCT05127434), human cytomegalovirus (NCT05085366), ZIKV (NCT04917861), rabies virus (NCT02241135, NCT03713086), Epstein-Barr virus (NCT05164094), Nipah virus (NCT05398796), Chikungunya virus (CHKV) (NCT03829384), human metapneumovirus and human parainfluenza (NCT04144348). In addition to vaccines for infectious diseases, mRNA is now being employed for various immunotherapeutic applications, including arming immune cells with antigen receptors and in vivo production of therapeutic antibodies or immunomodulator proteins. Of note, mRNA-based immunomodulators have successfully entered clinical trials. Moreover, mRNA can be used to improve the safety profile of CAR-T cell therapy and to allow for concurrent modification of lymphocytes by co-delivery of multiple mRNAs. In the future, delivery of multifunctional drug treatments with targeting mRNA-LNPs may further improve prevention and treatment of many diseases.

Cancer is major target for mRNA vaccines, as customized vaccines can be applied to target tumor neoantigens of individual patients. Current examples are BNT-122 (autogene cevumeran, RO7198457) developed by BioNTech and Genentech (NCT04486378, NCT04813627), and mRNA-4157 by Moderna (NCT03897881). Advancements in next-generation sequencing and AI computation have also made it possible to identify ideal neoantigens, which brings the goal of personalized neoantigen-based mRNA cancer vaccines within reach [258]. mRNAs that encode tumor antigens can also be used to generate DC-based vaccines [23] and CAR-T cells ex vivo as well as in vivo. Along these lines, BioNTech developed an mRNA-based immunotherapy (BNT211) for Claudin-6-positive solid tumors (NCT04503278) in which an mRNA lipoplex encoding CAR-T target antigens was administered to the patient. This treatment was able to sustain expansion and persistence of functional CAR-T cells in vivo [134]. Along with treating cancers, mRNA-derived CAR-T cells can potentially be used to treat other diseases. For example, CD5-targeted mRNA-LNPs were used to generate CAR-T cells in vivo in a mouse model of heart disease. The CAR-T cells reduced fibrosis and restored cardiac function in the animals [180], demonstrating a novel therapeutic use of mRNA-based drugs.

Another therapeutic application of mRNA is the use of cells as factories to produce functional proteins for protein-replacement therapies. This approach has been explored in treatment of cardiac disease [135], lung disease [184], genetic metabolic disease [24], hepatic disease [222], orthopedic disorders [15], neurodegenerative disorders [147], and muscle atrophy [195]. Though most studies are still in preclinical stages, mRNA drugs encoding a vascular endothelial growth factor (NCT03370887) and a cystic fibrosis transmembrane conductance regulator (NCT03375047) have entered clinical development.

Beside supplementing cells with functional proteins, mRNA can also be used to deliver antibodies that protect from infectious diseases, such as HIV-1 [152], RSV [218], CHKV [112] or ZIKV [55]. mRNAs can also encode antibodies that stimulate the immune system to kill tumors [181, 207, 217] as well as immune stimulatory factors [56] or ligands [75] to modulate the tumor microenvironment. Furthermore, mRNA drugs can be used to deliver gene editors, such as zinc finger nucleases (ZFNs), transcription activator-like effector nucleases (TALENs), and the clustered regularly interspaced short palindromic repeat (CRISPR)-associated protein (CRISPR/Cas) nucleases [271]. With all of these potential uses, mRNA is expected to become one of the major pillars of drug development in the next decade.

Merely being used as prophylactic vaccines, mRNA-based drugs already occupied a dramatic share of drug market value in the past years, as demonstrated by the revenue of BNT/Pfizer’s BNT162b2 (Comirnaty) ranked as the top ($36.8 billion) and Moderna’s Spikevax ranked as the 3^rd^ ($17.7 billion) in 2021 [52], as well as respectively showing slight growth to $37.8 billion [110] and $18.4 billion [51] in 2022. With such wide-ranging applications, the aggregate market value of mRNA vaccines and therapeutics is anticipated to grow to more than US$100 billion in 2029 [231] and more than US$120 billion in 2032 [163]. Taking advantage of mRNA-based drugs, the global AI market for vaccine development was $8.3 billion in 2022 with a market capitalization of $118.69 billion to be projected by 2030 [235], due to raising occurrence of emerging infectious diseases and extended applications to cancers as anticipated.

The first class of RNA-based drugs to gain approval by regulatory agencies was antisense oligonucleotides (ASOs), with Eteplirsen and Nusinersen approved by the US FDA in 2016. The next class to be approved was siRNAs in 2018 (Patisiran). Then in 2020, the mRNA vaccines tozinameran and elasomeran were approved. Consensus annual global sales forecasts of the 12 regulatory agency-approved RNA drugs were extracted from the GlobalData Intelligence Center database (Fig. 6B–D), and the forecasts for each drug up to 2029 are shown in Fig. 6E–G. While the forecasts are high, many factors contribute to the actual sales volume and annual growth of a drug, including unmet need/burden of the disease, clinical efficacy, comparators, safety and price [196]. Nevertheless, the highly promising clinical applications and market values of RNA therapeutics are expected due to reported revenues from currently launched drugs. Furthermore, fast growing market shares of RNA therapeutics are anticipated, as more than 720 mRNA and interfering/modulating RNA candidate therapeutics for many medical conditions are under development by companies and academic institutions worldwide.

Continued research and development on mRNA-based drugs will be best served by making use of AI technology and advanced high-throughput and high-speed technologies. These technologies can help in the design, selection and validation of DNA template, sequence composition, structural antigen features, chemical modification, formulation, delivery system, and manufacturing process. Optimization of these factors can then benefit mRNA translation efficiency, purity, cellular stability, non-immunogenicity, non-toxicity, thermostability, cellular uptake efficiency, organ-specific targeting ability, control activation of immune system, pharmacokinetics and pharmacodynamics (PK/PD), and cost-effectiveness. All of these factors will be the focus of future research projects and can open new opportunities for academic and industrial groups, especially those further strengthened with multidisciplinary collaborations. Through continued advancements, the full potential of mRNA vaccines and therapeutics can be realized in the twenty-first century and bring great benefit to human health and quality of life worldwide.

## Data Availability

All the data and materials supporting the conclusions were included in the main paper.
